# Characterization of mutant versions of the R-RAS2/TC21 GTPase found in tumors

**DOI:** 10.1038/s41388-022-02563-9

**Published:** 2022-12-07

**Authors:** Laura Clavaín, Isabel Fernández-Pisonero, Nieves Movilla, L. Francisco Lorenzo-Martín, Blanca Nieto, Antonio Abad, Rósula García-Navas, Clara Llorente-González, Manuel Sánchez-Martín, Miguel Vicente-Manzanares, Eugenio Santos, Balbino Alarcón, José M. García-Aznar, Mercedes Dosil, Xosé R. Bustelo

**Affiliations:** 1 Molecular Mechanisms of Cancer Program, Centro de Investigación del Cáncer, CSIC and University of Salamanca, 37007 Salamanca, Spain; 2 Instituto de Biología Molecular y Celular del Cáncer, CSIC and University of Salamanca, 37007 Salamanca, Spain; 3 Centro de Investigación Biomédica en Red de Cáncer (CIBERONC), CSIC and University of Salamanca, 37007 Salamanca, Spain; 4 Aragon Institute of Engineering Research, Department of Mechanical Engineering, University of Zaragoza, 50018 Zaragoza, Spain; 5 Transgenesis Facility and Nucleus Platform for Research Services, University of Salamanca, 37007 Salamanca, Spain; 6 Centro de Biología Molecular Severo Ochoa, CSIC and Universidad Autónoma de Madrid, 28049 Madrid, Spain

**Keywords:** Oncogenes, Cancer models, Focal adhesion, Cell signalling

## Abstract

The R-RAS2 GTP hydrolase (GTPase) (also known as TC21) has been traditionally considered quite similar to classical RAS proteins at the regulatory and signaling levels. Recently, a long-tail hotspot mutation targeting the R-RAS2/TC21 Gln^72^ residue (Q72L) was identified as a potent oncogenic driver. Additional point mutations were also found in other tumors at low frequencies. Despite this, little information is available regarding the transforming role of these mutant versions and their relevance for the tumorigenic properties of already-transformed cancer cells. Here, we report that many of the *RRAS2* mutations found in human cancers are highly transforming when expressed in immortalized cell lines. Moreover, the expression of endogenous R-RAS2^Q72L^ is important for maintaining optimal levels of PI3K and ERK activities as well as for the adhesion, invasiveness, proliferation, and mitochondrial respiration of ovarian and breast cancer cell lines. Endogenous R-RAS2^Q72L^ also regulates gene expression programs linked to both cell adhesion and inflammatory/immune-related responses. Endogenous R-RAS2^Q72L^ is also quite relevant for the in vivo tumorigenic activity of these cells. This dependency is observed even though these cancer cell lines bear concurrent gain-of-function mutations in genes encoding RAS signaling elements. Finally, we show that endogenous R-RAS2, unlike the case of classical RAS proteins, specifically localizes in focal adhesions. Collectively, these results indicate that gain-of-function mutations of R-RAS2/TC21 play roles in tumor initiation and maintenance that are not fully redundant with those regulated by classical RAS oncoproteins.

## Introduction

R-RAS2 (originally known as teratocarcinoma clone 21 or TC21) received significant attention upon its discovery in 1990 [1, 2] since it was considered a potential surrogate oncogenic pathway for cancer cells lacking gain-of-function mutations in the classical RAS GTPases (H-RAS, K-RAS, N-RAS; referred to hereafter as RAS proteins). This idea was based on several features of R-RAS2 that were unique among the rest of RAS superfamily members: (i) 100% homology at the protein level of the two effector regions (switch I and switch II) with those present in RAS proteins [1]; (ii) binding to a similar spectrum of RAS regulators and proximal effectors such as PI3K, the RAL GDP dissociation stimulator (RAL GDS), and RAF family members [3–10]; (iii) similar subcellular localization as RAS proteins [3–5]; and (iv) transforming activity of some of gain-of-function R-RAS2 mutant versions similar to (or even higher than) RAS oncoproteins [2, 11]. Despite these data, the interest in this GTPase faded away subsequently due to the inability to find gain-of-function *RRAS2* mutations in human tumors with the techniques available before the emergence of next-generation sequencing.

Recent observations and technological advances have now led to a renewed interest in this GTPase. The extensive sequencing of cancer genomes during this last decade revealed that the *RRAS2* locus harbors long-tail hotspot mutations (namely, Q72L and Q72H) [12, 13]. This Gln^72^ residue is analogous to the Gln^61^ found in RAS proteins (the shift in numbers is due to the presence of an additional 11-amino-acid N-terminal sequence in the primary structure of R-RAS2). Mutations in this Gln^72^ position are known to induce a gain-of-function oncogenic effect, as they impair the hydrolysis of the bound GTP that maintains these GTPases in the active state [14, 15]. Other *RRAS2* mutations analogous to those targeting the residues Gly^12^, Gly^13^ or Ala^59^ of RAS protein residues have also been detected in pan-cancer studies at very low frequencies (www.cbioportal.org). In addition, *RRAS2* mutations have been found at higher frequencies in central nervous system germinomas (in 14.6% of interrogated cases) and KIT^+^ germ cell tumors (in 11.8% of cases) [16, 17]. Highlighting the pathogenic importance of the *RRAS2*^Q72L^ mutation, we have recently used an inducible knock-in mouse strain to show that somatic expression of the encoded mutant protein drives the rapid development of many tumor types [18]. *RRAS2* missense mutations, including Q72L, have been also identified in Noonan syndrome [19, 20], a rare developmental disease frequently associated with the deregulation of RAS signaling elements [21, 22]. Evidence obtained with mouse models also indicates that the wild-type version of R-Ras2 favors pro-tumorigenic processes activated by other oncogenes. Thus, we have shown that the elimination of endogenous R-Ras2 leads the abrogation of Her2-driven mammary tumorigenesis as well as of Notch1-driven T cell acute lymphoblastic leukemia [18, 23]. Conversely, the overexpression of R-Ras2 per se promotes the development of a B-cell chronic lymphocytic leukemia-like condition in mice [24]. R-RAS2 also plays roles in normal physiological conditions, including in mammary gland development, the negative selection of T lymphocytes, B-cell–dependent germinal responses, T cell phagocytosis and T cell receptor homeostatic signaling [25–30].

Despite this recent progress, the role of R-RAS2 in tumorigenic processes is still obscure. For example, it is unclear whether it plays fully redundant roles with RAS oncoproteins. While the overlap in cell signaling suggests that some redundancy might exist, results from R-Ras2^Q72L^-expressing mice have revealed that this mutant protein induces a spectrum of tumor types that is not redundant with those generated by oncogenic Ras proteins [18]. Further, ectopic expression of R-Ras2 cannot rescue the proliferative defect caused by the elimination of the three Ras proteins in mouse embryonic fibroblasts, arguing against a fully functional overlap of these proteins [31]. Another issue that remains unsolved is the role of the oncogenic versions of R-RAS2 in already-transformed cells. In line with this, we have shown that the endogenous R-Ras2^Q72L^ mutant is critical for maintaining the transformed phenotype of fibrosarcoma cell lines derived from R-Ras2^Q72L^-expressing mice [18]. However, it is unclear whether this essentiality is maintained in human cancer cell lines that accumulate a larger number of genomic alterations, which can also entail gain-of-function mutations in RAS pathway genes. It is also worth noting that most work with R-RAS2 has been done using ectopic systems that can obscure or provide inaccurate information about the actual function of the endogenous proteins.

To tackle these unresolved issues, we have now undertaken several intertwined research avenues. On the one hand, we cataloged additional *RRAS2* mutations found in human tumors to identify those that are most likely to be pathogenic. On the other hand, we analyzed the role of the endogenous R-RAS2 protein in human cancer cells. Our results indicate that many mutations found in human tumors are likely to be oncogenic, namely due to the engagement of the PI3K–AKT pathway. They also show that endogenous R-RAS2 has a subcellular localization that is distinct from that of RAS proteins. Finally, we found that the expression of endogenous R-RAS2^Q72L^ is essential to maintain key biological features of cancer cells. Remarkably, maintaining these key functions is relevant even in cells that harbor mutations in RAS signaling elements. Collectively, these data indicate that oncogenic R-RAS2 plays important and specific roles in cancer cells.

## Results

### Some *RRAS2* gain-of-function mutations found in tumors are oncogenic

**Fig. 1:**
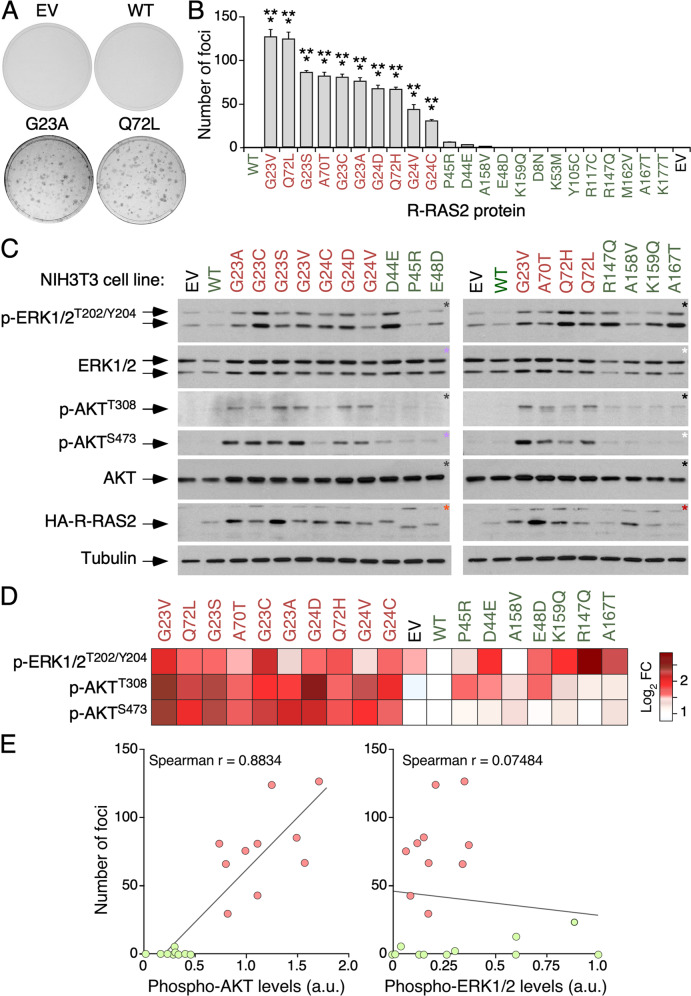
Oncogenic activity of *RRAS2* mutations found in tumors. **A** Representative images of stained tissue culture plates at the end of the focus formation assays with the indicated R-RAS2 proteins. EV empty vector; WT wild-type. **B** Transforming activity of the indicated R-RAS2 mutant proteins. Data represent mean ± SEM (*n* = 3). Statistical significance was tested by one-way ANOVA and Dunnett’s multiple comparison tests (***p < 0.001). Non-transforming and transforming mutants are shown in green and red, respectively. **C** Representative immunoblots showing the levels of phosphorylation and total amount of indicated proteins (left) in lysates obtained from NIH3T3 cells stably expressing the described proteins (top) after starvation for four hours. Aliquots from the same lysates were analyzed in separate blots (each identified with asterisks of the same color). Levels of tubulin α were used as a loading control (*n* = 3). p, phosphorylated. **D** Heatmap illustrating the phosphorylation levels of the indicated proteins (left) obtained in **C**. Phosphorylation levels were normalized to total protein levels. Data are represented as the fold-change (FC) variation as compared to the wild-type protein using a gradient color according to the scale shown on the right. **E** Correlation of transforming activities and phospho-AKT (left) and phospho-ERK (right) triggered by the R-RAS2 mutants. Values were normalized (from 0 to 1) for representation purposes. Phospho-AKT levels were calculated by combining the signals of phospho-AKT^T308^ and phospho-AKT^S473^. Spearman correlation coefficient (Spearman r) is indicated for each analysis inside the appropriate graph.

The Q72L, G23V and Dup24-26 *RRAS2* mutations, analogous to the Q61L, G12V and Dup13-15 mutations in RAS proteins [22], can activate the transforming function of R-RAS2 in cell culture [2, 11, 32, 33]. We have also recently shown that the *Rras2*^Q72L^ mutation acts as a potent oncogenic driver when somatically expressed in mice [18]. Based on this, we tested the transforming activity of a collection of 21 additional *RRAS2* mutations that are found in human tumors at very low frequency, according to data from the cBioPortal database. Of note, all *RRAS2* mutations targeting the residues Gly^23^ (G23A/C/S/V), Gly^24^ (G24C/D/V), Ala^70^ (A70T) or Gln^72^ (Q72L/H) display transforming activity in focus formation assays (Fig. 1A, B). The strongest activity was observed for the G23V and Q72L mutants, followed by six mutations targeting Gly^23^ (G23A/C/S), Gly^24^ (G24D), Ala^70^ (A70T) or Gln^72^ (Q72H), and finally, by two mutations that modify the Gly^24^ residue (G24C/V) (Fig. 1B). Very marginal transforming activity was found for the P45R, D44E or A158V R-RAS2 mutants (Fig. 1B). No detectable transforming activity was observed for the wild-type or the remaining mutant versions of R-RAS2 in these assays (Fig. 1A, B). These data indicate that, like mutations of classical RAS proteins [22], the mutations targeting the Gly^23^, Gly^24^, Ala^70^ or Gln^72^ residues of R-RAS2 are oncogenic.

To compare the signaling of both the transforming and non-transforming mutants, we analyzed the phosphorylation status of both ERK1/2 and AKT in total cellular extracts obtained from serum-starved NIH3T3 cells stably expressing some of these R-RAS2 mutant versions. Using immunoblot analyses, we found that all oncogenic R-RAS2 proteins could concurrently activate the ERK and PI3K–AKT pathways to either moderate or high levels (Fig. 1C, D; mutations shown in red). In contrast, the non-transforming mutants marginally stimulated these two pathways (e.g., P45R, E48D and A185V) or only activated the ERK1/2 route (e.g., D44E, R147Q, K159Q and A167T) (Fig. 1C, D; mutations shown in green). Interestingly, the correlation between the transforming and signaling activities of all interrogated mutants suggests that the oncogenic activity of R-RAS2 is directly linked to the ability to engage the PI3K–AKT axis (Fig. 1E).

### Endogenous R-RAS2^Q72L^ is required for tumorigenesis of cancer cells

**Fig. 2:**
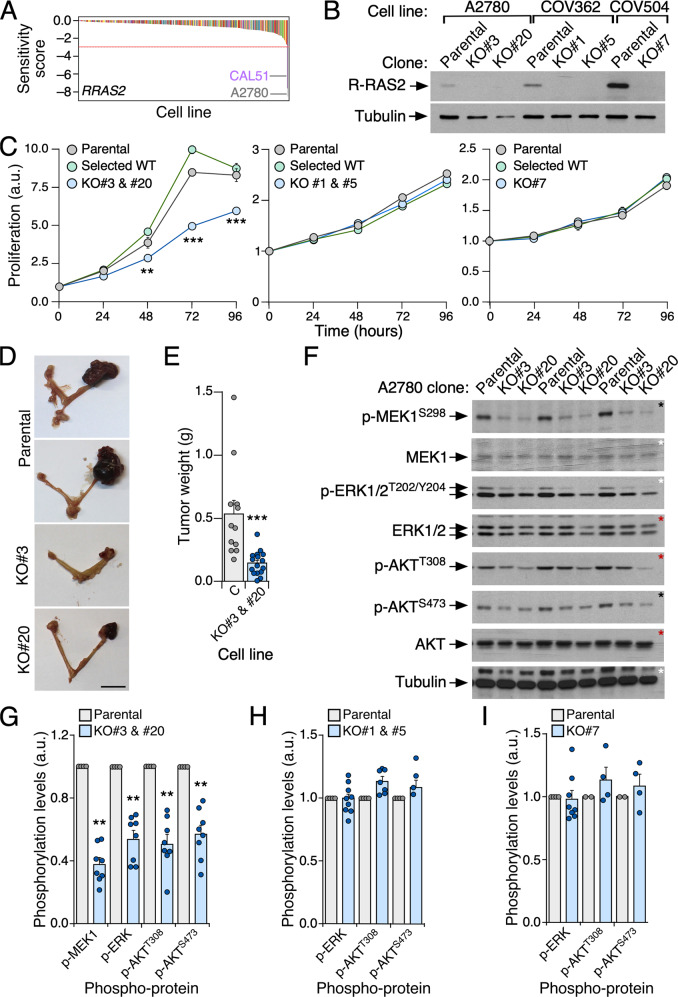
Endogenous R-RAS2^Q72L^ is required for tumorigenesis of cancer cells. **A** Plot illustrating the R-RAS2 dependency of cancer cell lines using the data contained in the Novartis Drive Data Portal. The name of R-RAS2-dependent cell lines whose proliferation is above the minimal sensitivity score threshold (red line) is shown on the graph. **B** Representative immunoblot analysis showing the expression of indicated proteins (left) in lysates from the cells shown at the top. KO#3, KO#20, KO#1, KO#5 and KO#7 indicate independent clones of *RRAS2* knockout (KO) cells. Levels of tubulin α were used as a loading control (*n* = 3). **C** Proliferation of indicated A2780 (left), COV362 (middle) and COV504 (right) control and derivative cells (insets) using MTT assays. “Selected WT” indicate cell clones that went through all to the CRISPR–Cas9 gene editing process but that have not lost the *RRAS2* gene. Data represent mean ± SEM (*n* = 3). Statistical significance was tested by two-way ANOVA and Tukey’s multiple comparison tests (**p < 0.01; ***p < 0.001). **D** Representative images of tumors generated by indicated A2780 cell lines (left) upon implantation into the ovarian bursa of immunocompromised mice. Scale bar, 1 cm. **E** Quantitation of the tumors obtained in **D**. Points represent results from an individual mouse. Bars represent the mean ± SEM. *n* = 12 for controls; *n* = 16 for *RRAS2*-deficient clones #3 and #20 (which were pooled together). Statistical significance was tested by Mann–Whitney test (***p < 0.001). **F** Representative immunoblots showing the phosphorylation and total amount of indicated proteins (left) in lysates from the cells indicated at the top. Aliquots from the same lysates were analyzed in separate blots (each identified with asterisks of the same color). Levels of tubulin α were used as a loading control (*n* = 3). **G** to **I** Quantitation of phosphorylation levels of the described proteins (bottom) expressed in the indicated A2780 (**G**), COV362 (**H**) or COV504 (**I**) cell line derivatives and their appropriate controls (top). Bars represent mean ± SEM. *n* = 4 (for **G**); *n* = 3 (for **H** and **I**). For (**G**), statistical significance was tested by Mann–Whitney test (**p < 0.01).

As the somatic expression of the endogenous R-Ras2^Q72L^ promotes tumor formation in mice [18], we decided to investigate whether the endogenous expression of R-RAS2^Q72L^ is also important for maintaining the transformed phenotype of already-established human cancer cell lines. As a first approximation, we used the Novartis Drive Data Portal [34] to identify cell lines that are vulnerable to the shRNA-mediated depletion of either the wild-type or oncogenic mutants of R-RAS2. Of the 387 cell lines in that dataset, only the proliferation of the A2780 ovarian cell line and the CAL-51 breast cancer cell line showed R-RAS2-dependency (Fig. 2A). Of note, both cell lines have a *RRAS2*^Q72L^ mutation [2, 35]. As a control, we identified several cell lines (excluding A2780 and CAL-51) whose proliferation is dependent on K-RAS (100 cell lines), N-RAS (25 cell lines) or H-RAS (26 cell lines) (Supplementary Fig. 1A). We did not identify R-RAS- or M-RAS-dependent cell lines in this dataset (Supplementary Fig. 1B). To confirm these in silico observations, we used CRISPR–Cas9 gene editing to knockout the *RRAS2* locus from A2780 cells (R-RAS2^Q72L^ positive), two ovarian cancer cell lines that express the wild-type version of R-RAS2 (COV362 and COV504) and the breast CAL-51 cancer cell line (which is R-RAS2^Q72L^ positive). Effective knockout of the *RRAS2* gene in each of these cell lines was confirmed by genomic DNA sequencing (see Materials and Methods) and immunoblot analyses (Fig. 2B and Supplementary Fig. 1C). The knockout of endogenous R-RAS2^Q72L^ in A2780 cells reduced the rates of cell proliferation, while the knockout of wild-type R-RAS2 in COV362 or COV504 cells did not (Fig. 1C). No differences in proliferation rates were seen for control A2780 cells subjected to CRISPR–Cas9 gene editing and subsequent cloning steps (but without knocking out *RRAS2*) (Fig. 1C). Consistent with these data, we observed that the elimination of endogenous R-RAS2^Q72L^ also reduces the tumor-forming activity of A2780 cells when orthotopically implanted in the ovarian bursa (Fig. 1D, E). Likewise, elimination of endogenous R-RAS2^Q72L^ from CAL-51 cells (Supplementary Fig. 1C) reduced their proliferation in cell culture (Supplementary Fig. 1D) and in vivo transforming activity when implanted in the mammary gland pad (Supplementary Fig. 1E, F). Importantly, A2780 and CAL-51 cells harbor extra-mutations in *PIK3CA* (E365K/+ in A2780 cells, and E542K/+ in CAL-51 cells), *BRAF* (V22M/+ in A2780 cells) and/or *RAF1* (A629S/+ in CAL-51 cells). This indicates that RRAS2^Q72L^ is relevant for the transforming activity of cancer cells even when they harbor gain-of-function mutations in classical signaling elements for both R-RAS2 and RAS pathways.

The *RRAS2*^Q72L^ knockout also led to statistically significant reductions in the phosphorylation levels of signaling elements of the MEK–ERK1/2 and PI3K–AKT pathways, both in A2870 cells (Fig. 2F, G) and CAL-51 cells (Supplementary Fig. 1G). Taken together, these data indicate that the endogenous expression of *RRAS2*^Q72L^ is important for the fitness of cancer cells as well as for the activation of the ERK and AKT pathways, even when cells contain co-mutations in RAS and R-RAS2 signaling elements. In contrast, we did not find those signaling defects in wild-type R-RAS2-deficient COV362 or COV504 cells (Fig. 2H, I).

### Endogenous R-RAS2 proteins localize in focal adhesions

**Fig. 3:**
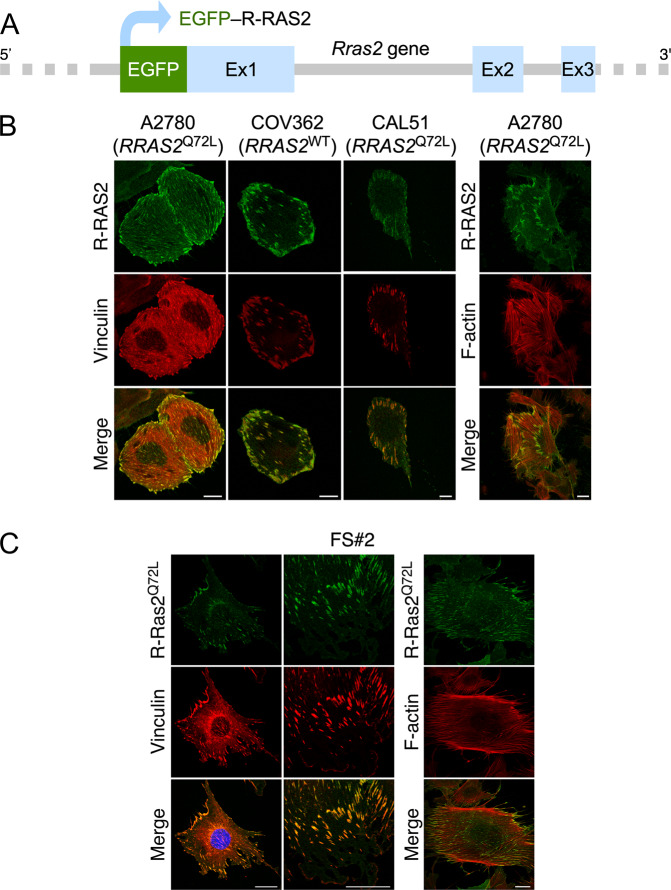
Endogenous R-RAS2 proteins localize in focal adhesions. **A** Schematic representation of the *RRAS2* locus upon the CRISPR–Cas9 gene editing step described in the main text. **B** and **C** Representative confocal microscopy images of indicated GFP-tagged cell lines (**B** and **C**, top) after staining with antibodies to vinculin (left panels) or with phalloidin (**B** and **C**, two columns of panels on the right). Scale bars, 10 μm (human cells) and 25 μm (fibrosarcoma cells).

Previous reports using ectopic expression systems have shown that R-RAS2 displays a subcellular localization highly similar to RAS proteins [3–5, 19, 22]. To determine whether the endogenous protein also has this subcellular localization, we used CRISPR–Cas9 gene editing to insert an EGFP-encoding DNA sequence in-frame with the *RRAS2* open reading frame in the first exon of the *RRAS2* locus in the A2780, CAL-51 and COV362 cell lines (Fig. 3A). In addition, we performed an analogous gene editing step in a primary cancer cell line (FS#2) generated from a fibrosarcoma that was induced upon the somatic expression of endogenous R-Ras2^Q72L^ in mice [18]. With this strategy, we could easily visualize the endogenous R-RAS2 protein in those cells using confocal epifluorescence microscopy. In contrast to the *RRAS2* knockout in A2780 cells (see above, Fig. 2C) or in the fibrosarcoma #2 cell line [18], we did not detect any alteration in the proliferative rates of the cell line derivatives expressing the EGFP-tagged version of endogenous R-RAS2^Q72L^ (LC, IF-P and XRB, unpublished data). This indicates that the attachment of the EGFP tag does not alter the function of the GTPase. Unlike previous results with the ectopic proteins, we did not observe any endogenous wild-type R-RAS2 and R-RAS2^Q72L^ proteins in the plasma membrane, Golgi apparatus or endoplasmic reticulum in any tested human cell line (Fig. 3B) or mouse cell line (Fig. 3C). Rather, we found that endogenous wild-type R-RAS2 and R-RAS2^Q72L^ proteins specifically localize in both nascent and mature focal adhesions in all cell lines tested (Fig. 3B, C). Consistent with this, the distribution of EGFP-R-RAS2 overlaps with the focal adhesion marker vinculin (Fig. 3B, C) and with the F-actin present in those structures (Fig. 3B, C). It is worth noting that that the EGFP-tagging per se could not be the cause of the subcellular localization of R-RAS2, given that endogenous EGFP-tagged K-RAS maintains its expected plasma membrane localization in cells [36]. These results indicate that, despite sharing a similar spectrum of downstream effectors, endogenous R-RAS2 and RAS proteins have different subcellular localization patterns.

### Endogenous R-RAS2^Q72L^ regulates focal adhesion- and invasion-related functions

**Fig. 4:**
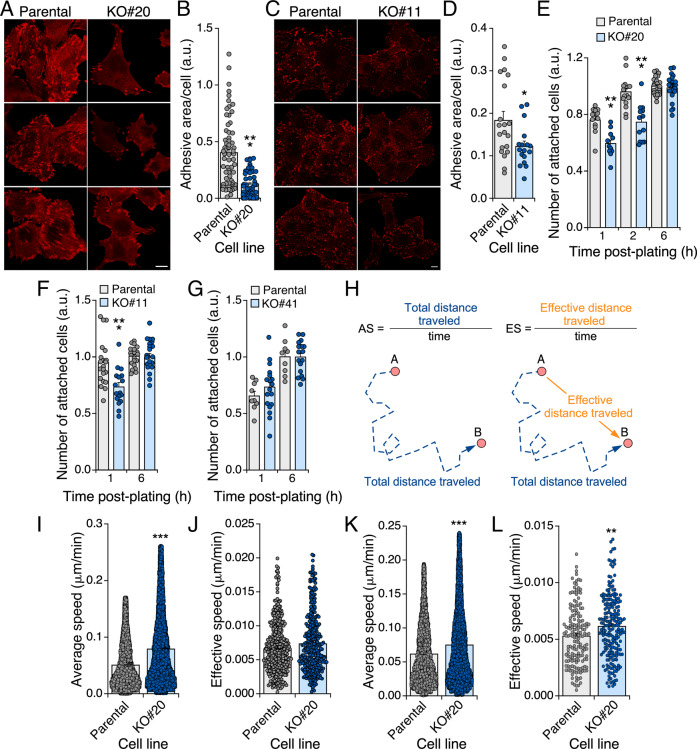
Endogenous R-RAS2^Q72L^ regulates focal adhesion-related functions. **A** Representative confocal microscopy images of parental and *RRAS2*^Q72L^ knockout A2780 cells (top) upon staining with antibodies to vinculin. Scale bar, 10 μm. **B** Quantitation of the total area of focal adhesions per cell from experiments shown in A. Points represent individual cells (*n* = 3 independent experiments). Bars represent mean ± SEM. Statistical significance was tested by the Mann–Whitney test (***p < 0.001). **C** Representative confocal microscopy images of parental and *RRAS2*^Q72L^ knockout CAL-51 cells (top) after staining with antibodies to vinculin. Scale bar, 10 μm. **D** Quantitation of the total area of focal adhesions per cell from the experiments shown in **C**. Points represent individual cells (*n* = 3). Bars represent mean ± SEM. Statistical significance was tested by the unpaired *t*-test (*p < 0.05). **E** to **G** Attachment of the cell lines A2780 (**E**), CAL-51 (**F**) or COV362 (**G**) and the appropriate control cells (top) at the indicated times after plating (bottom). Data were normalized using the number of cells initially seeded (counted at 6 h after plating). Points represent individual cells (*n* = 3). Bars represent mean ± SEM. Statistical significance was tested by two-way ANOVA analysis and Sidak’s multiple comparisons tests (***p < 0.001). **H** Scheme and formula of the two migration-related parameters utilized in **I** to **L**. AS average speed, ES effective speed. The migration of cells is shown as a broken blue lane. **I** and **J** Average (**I**) and effective (**J**) speed of the indicated A2780 cells (bottom) in collagen-based hydrogels. Points represent individual cells (*n* = 3). Bars represent mean ± SEM. Statistical significance was tested by the Mann–Whitney test (***p < 0.001). **K** and **L** Average speed (**K**) and effective speed (**L**) of the indicated A2780 cells (bottom) in collagen-based hydrogels with a gradient of insulin growth factor 1. Points represent individual cells (*n* = 3). Bars represent mean ± SEM. Statistical significance was tested by the Mann–Whitney test (***p < 0.001).

Based on these observations, we next investigated whether R-RAS2 is involved in focal adhesion-related functions using 2D cultures. We first compared the adhesive area of parental and *RRAS2*-knockout A2780 and CAL-51 cells by confocal microscopy after staining with antibodies to vinculin. Lack of endogenous R-RAS2^Q72L^ impaired mature focal adhesion formation in both A2780 cells (Fig. 4A, B) and CAL-51 cells (Fig. 4C, D) as compared to their respective controls. In sharp contrast, elimination of the two wild-type alleles of the *RRAS2* gene had no overt effects on the adhesive area in COV362 cells (Supplementary Fig. 2A, B). Consistent with these observations, both A2780 cells (Fig. 4E) and CAL-51 cells (Fig. 4F) lacking endogenous R-RAS2^Q72L^ exhibited transient defects in adhesion. This defect was not observed for COV362 cells lacking wild-type R-RAS2 (Fig. 4G).

We next compared the ability of control and *RRAS2*^Q72L^-knockout A2780 cells to invade a 3D-matrix environment. For this, we embedded cells into collagen hydrogels contained in microfluidics chambers and, subsequently, quantified by cell microscopy their average speed (total distance traveled per unit of time) (Fig. 4H, left) and effective speed (effective distance traveled per unit of time) (Fig. 4H, right). Note that effective speed indirectly gives information about the directionality of cell movements (Fig. 4H, right). We observed that the R-RAS2^Q72L^-depleted A2780 cells show an increase in average speed as compared to controls (Fig. 4I), while their effective speed remains unchanged (Fig. 4J). These data indicate that they move faster but do not change the randomness of their movements. Those two parameters change more coordinately when the *RRAS2*^Q72L^-deficient cells are subjected to a chemotactic gradient of insulin growth factor 1 (IGF1) in the microfluidics chambers (Fig. 4K, L). Wild-type R-RAS2-deficient COV362 cells showed a slight decrease in both the average (Supplementary Fig. 2C) and effective (Supplementary Fig. 2D) speed as compared to controls. This suggests that, at least for the cell lines tested in this study, the impact of this GTPase on focal adhesion- and invasion-related functions is dependent on the mutational status of the *RRAS2* gene in cancer cells. As it will be shown below (Fig. 6), it is likely that the 3D properties exhibited by R-RAS2^Q72L^-knockout cells are influenced by other invasion-related parameters in addition to focal adhesion numbers such as, for example, the differential expression of integrins and extracellular matrix remodeling factors.

### Impact on the transcriptome of endogenous R-RAS2^Q72L^

**Fig. 5:**
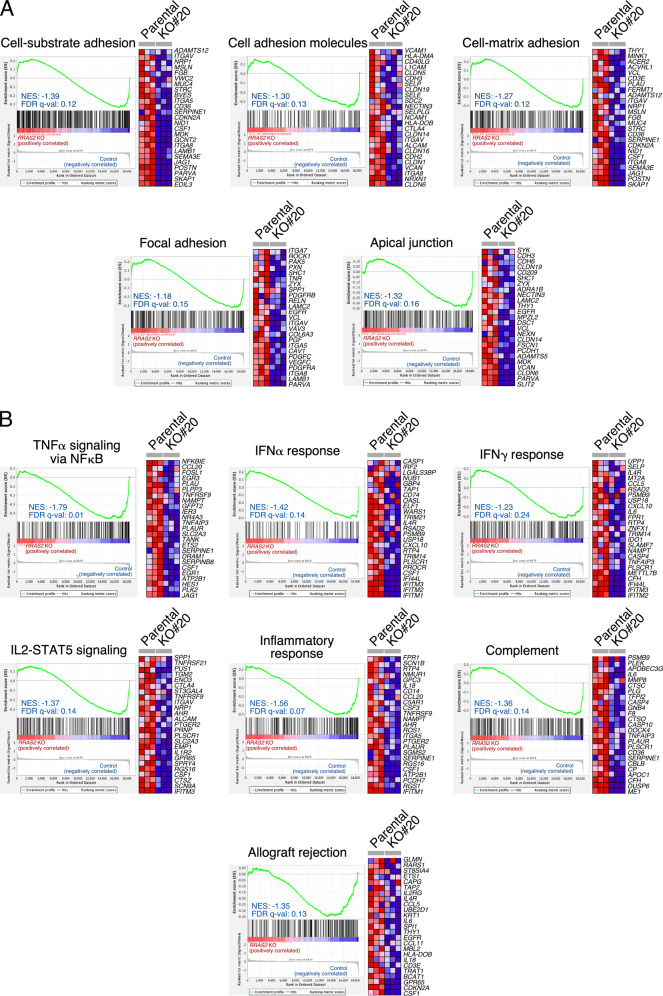
Impact on the transcriptome of endogenous R-RAS2^Q72L^. **A** and **B** Examples of gene signatures enriched in the downregulated transcriptome of *RRAS2*^Q72L^ knockout A2780 cells. The FDR *q* value and NES (normalized enrichment score) for the downregulated gene signatures are indicated in each plot. In both cases, GSEA analyses were conducted using selected gene signatures for adhesion-related processes (**A**) and the unbiased pathways contained in the MSigDB hallmark collection (**B**).

To get a more general view of the impact of R-RAS2^Q72L^ signaling on the transformed phenotype of cancer cells, we next analyzed control and R-RAS2^Q72L^-deficient A2780 cells using microarray analyses. We found that the elimination of endogenous R-RAS2^Q72L^ led to the consistent downmodulation (313) and upregulation (287) of specific gene subsets (Supplementary Fig. 3A, B and Supplementary Table 1). Using standard gene annotation methods, we found that the downregulated transcriptomal subset of the knockout cells includes genes primarily associated with chemotaxis-, cell-migration, and cell adhesion functions (Supplementary Table 2). Consistent with this, we found using gene set enrichment analysis (GSEA) that the downregulated transcriptome found in the *RRAS2*^Q72L^ knockout cells was enriched in gene signatures connected to the foregoing functions (Fig. 5A). This indicates that R-RAS2^Q72L^ is likely to have not only a specific role in focal adhesion formation and/or stability but also a broader role in the regulation of the expression of proteins implicated in cell adhesion processes. Consistent with the downregulation of p-ERK and p-AKT signaling detected in our signaling experiments (see above, Fig. 2F, G), we also found a reduction in the expression of gene signatures associated with K-RAS signaling (Supplementary Fig. 3C) and the downregulation of negative regulators of the MEK pathway (Supplementary Table 2). Other downregulated gene signatures in R-RAS2^Q72L^-knockout A2780 cells identified using GSEA included those associated with inflammatory and immune processes, such as those linked to the stimulation of the tumor necrosis factor α (TNFα)–nuclear factor κB (NFκB) axis, the interferon (IFN) α and γ pathways, the interleukin 2–Stat5 route as well as those connected to biological responses influenced by those signaling pathways (e.g., inflammation, complement, allograft rejection) (Fig. 5B).

According to standard functional annotation analyses, the upregulated transcriptome present in *RRAS*2^Q72L^ knockout A2780 cells contained functional subsets composed of very few genes, although those with smaller *p* values were associated with blood vessel formation, collagen catabolic processes (metalloproteinases, collagen chains), and a variety of unconnected developmental processes (Supplementary Table 3). Further GSEA revealed the enrichment of genes signatures associated with DNA repair, G_2_/M-associated processes (ultraviolet ray responses, mitotic spindle, G2/M checkpoint), myogenesis, and PI3K–mTORC signaling (Supplementary Fig. 3D). The latter signature can be the result of the activation of compensatory mechanisms to overcome the deficient stimulation of this pathway in R-RAS2^Q72L^-deficient cells (see above, Fig. 2F, G). Finally, we have found that gene signatures associated with several metabolic pathways were found either downregulated (glycolysis) or upregulated (oxidative phosphorylation, heme metabolism, bile acid metabolism) in the R-RAS2^Q72L^ knockout cells when compared to controls (Supplementary Fig. 3E).

**Fig. 6:**
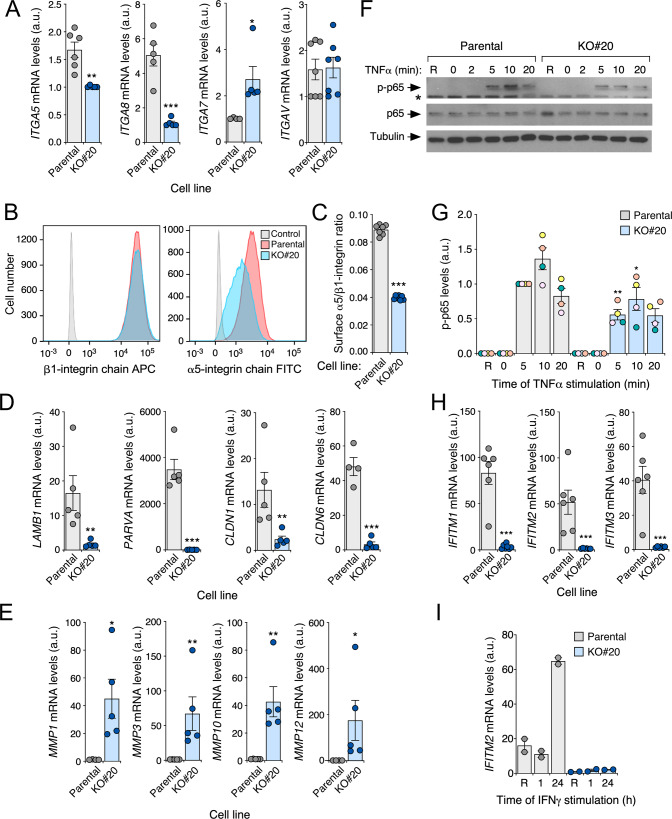
Validation of microarray data. **A** Expression of the indicated integrin-encoding transcripts in parental and *RRAS2*^Q72L^-knockout A2780 (clone KO#20) cells using qRT-PCR analyses. Points represent independent experiments. Bars represent mean ± SEM. Values are given in arbitrary units, taking the lowest mean of each experiment as 1. Statistical significance was tested by the Student’s *t*-test (*, *p* < 0.05; **, *p* < 0.01; ***, *p* < 0.001). **B** and **C** Example (**B**) and quantitation (**C**) of the protein expression levels of indicated integrins in parental and *RRAS2*^Q72L^-knockout A2780 (clone KO#20) cells. In **A**, control means the staining with an isotype control antibody. In **B**, points represent independent experiments. Statistical significance was tested by the Student’s *t*-test (***p < 0.001). **D** and **E** Expression levels of indicated transcripts (**D** and **E**, left) in parental and *RRAS2*^Q72L^-knockout A2780 (clone KO#20) cells using qRT-PCR analyses. Points represent independent experiments. Bars represent mean ± SEM. Values are given in arbitrary units, taking the lowest mean as 1. Statistical significance was tested by the Student’s *t*-test (*p < 0.05; **p < 0.01; ***p < 0.001). **F** and **G** Representative immunoblot analysis (**F**, top panel) and quantitation (**G**) of the phosphorylation (p-) of the p65 NFκB subunit upon the stimulation of serum-starved parental and *RRAS2*^Q72L^-knockout A2780 (clone KO#20) cells with TNFα for the indicated periods of time. As control, we evaluated the level of total p65 (**F**, middle panel) and tubulin α (**F**, bottom panel) in each sample. R, resting cells. In **G**, points represent independent experiments (values for each independent experiment shown with points of different colors). Bars represent mean ± SEM. Values represent arbitrary densitometry units (with 1 assigned to value obtained in control cells stimulated with TNFα for 5 min). Statistical significance was tested by the Student’s *t*-test (*p < 0.05; **p < 0.01). **H** Expression of the indicated *IFNITM* transcripts in parental and *RRAS2*^Q72L^-knockout A2780 (clone KO#20) cells using qRT-PCR analyses. Points represent independent experiments. Bars represent mean ± SEM. Values are given in arbitrary units, taking the lowest mean of each experiment as 1. Statistical significance was tested by the Student’s *t*-test (***p < 0.001). **I** IFNγ-triggered stimulation of *IFITM2* transcript levels in parental and *RRAS2*^Q72L^-knockout A2780 (clone KO#20) cells using qRT-PCR analyses (*n* = 2 independent experiments).

Confirming the impact of endogenous R-RAS2^Q72L^ in cytoskeletal-related processes, we found using quantitative reverse-transcription-polymerase chain reactions (qRT-PCR) the downregulation of genes encoding integrin α5 and α8 in *RRAS2*^Q72L^-knockout A2780 cells (Fig. 6A). This was specific, since the transcript levels for other integrins (integrin αv, integrin α7) were not similarly regulated (Fig. 6A). The downmodulation of integrin α5 at the protein level was also confirmed in *RRAS*2^Q72L^ knockout A2780 cells by flow cytometry (Fig. 6B, C). Likewise, we corroborated using qRT-PCR the strong downregulation of transcripts encoding cytoskeletal proteins (*PARVA*), tight junction elements (*CLDN1*, *CLDN6*), extracellular matrix proteins (*LAMB1*), and metalloproteinases (*MMP1*, *MMP3*, *MMP10*, *MMP12*) in the cells lacking endogenous R-RAS2^Q72L^ (Fig. 6D, E). All this transcriptomal changes were R-RAS2^Q72L^-specific because, with the exception of the deregulation seen for *CLDN6* and *MMP12* transcript levels (Supplementary Fig. 4A), the knockout of the two wild-type *RRAS2* alleles in COV362 cells did not elicit the changes in mRNA levels previously detected in *RRAS*2^Q72L^ knockout A2780 cells (Supplementary Fig. 4A). Consistent with the downmodulation of TNFα-related gene signatures found in the microarray experiments (see above, Fig. 5B), we found using immunoblot experiments that TNFα-induced phosphorylation of the p65 NF-kB subunit was less efficient in *RRAS*2^Q72L^ knockout A2780 cells than in controls (Fig. 6F, G). This was not the case in wild-type R-RAS2-deficient COV362 cells (Supplementary Fig. 4B). Finally, we also observed using qRT-PCR experiments that the expression of IFN-regulated genes (*IFITM1*, *IFITM2*, *IFITM3*) was severely reduced in A2780 cells lacking R-RAS2^Q72L^ when compared to the wild-type counterparts (Fig. 6H). Again, such changes were not detected in the case of COV362 cells lacking the endogenous wild-type version of R-RAS2 (Supplementary Fig. 4C). IFITM (interferon-inducible transmembrane) proteins are small proteins localized at the plasma membrane and/or intracellular vesicles that confer resistance to viral infections and regulate a variety of adaptive system-related immune responses [37]. The upregulation of these genes seen in IFNγ-stimulated cells was also eliminated in the absence of endogenous RRAS2^Q72L^ (Fig. 6I). Importantly, no changes in the expression of transcripts encoding IFNs (*IFNA*, *IFNG*), receptors (*IFNR1*, *IFNR2*), and primary mediators (*TYK2*, *JAK*) were detected in *RRAS2*^Q72L^ knockout A2780 cells (Supplementary Table 1 and Supplementary Fig. S4D). These results give further support to the specific connection of R-RAS2^Q72L^ signaling with focal adhesion-, cell invasion-, and immune system-related processes.

### R-RAS2 consistently regulates mitochondrial activity in cancer cells

**Fig. 7:**
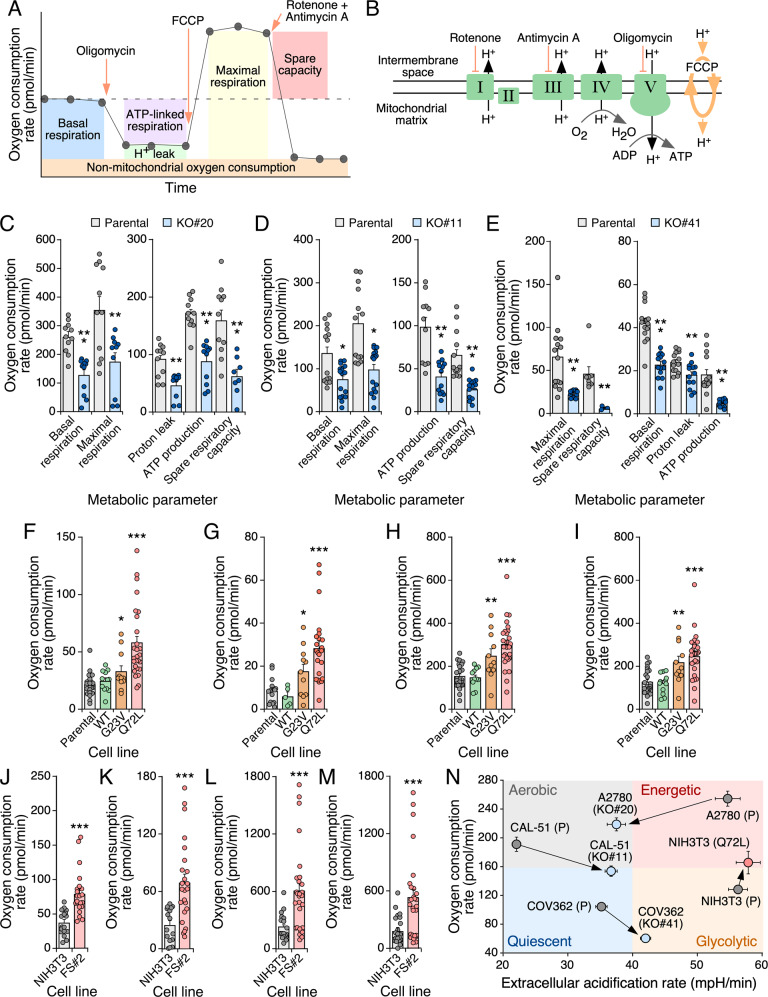
R-RAS2 regulates mitochondrial activity. **A** Schematic representation of the expected respiratory profile obtained using the Seahorse Mito Stress test. Metabolic parameters (boxes) were determined based on the OCR measurement following addition of the indicated drugs at the specified time points. **B** Depiction of the electron transport chain. I to V represent the complexes participating in the oxidative phosphorylation process. Inhibitors and targets used in the Seahorse tests are indicated. **C** to **E** Quantification of indicated metabolic parameters (bottom) in *RRAS2*^Q72L^ knockout A2780 (**C**), CAL-51 (**D**) and COV362 (**E**) cells and appropriate controls. Points represent independent experimental samples. Bars represent the mean ± SEM. *p < 0.05; **p < 0.01; ***p < 0.001 using unpaired t-test or Mann–Whitney test depending on the normality of each dataset (for *n*, see points on bars of each histogram). **F** to **I** Quantitation of the basal respiration (**F**), ATP production (**G**), maximal respiration (**H**), and spare respiratory activity (**I**) of NIH3T3 cells expressing the indicated R-RAS2 proteins (bottom) when compared to parental controls. Points represent independent experiments. Bars represent the mean ± SEM. *p < 0.05; **p < 0.01; ***p < 0.001 using one-way ANOVA and Sidak’s multiple comparisons tests (for *n*, see points on bars of each histogram). **J** to **M** Quantitation of basal respiration (**J**), ATP production (**K**), maximal respiration (**L**), and spare respiratory capacity (**M**) in NIH3T3 and *Rras2*^Q72L/Q72L^ fibrosarcoma FS#2 cells. Points represent independent experiments. Bars represent the mean ± SEM. ***p < 0.001 using Mann–Whitney test (for *n*, see points on bars of each histogram). **N** Energy map depicting the metabolic phenotype (color dot) and metabolic potential (arrow connecting each paired cell line) for indicated cell lines. Each quadrant in the graph represents the indicated metabolic phenotype. (P), parental; (KO#n), knockout clone #n; Q72L, ectopic R-RAS2^Q72L^. Data represent mean ± SEM. Data were not analyzed statistically (*n* = 6).

Given the changes in expression of gene signatures associated with oxidative metabolism and glycolysis found in *RRAS2*^Q72L^-deficient ovarian cancer cells (see above, Supplementary Fig. 3E), we next used the Seahorse analyzer (Fig. 7A, B) to investigate the status of these two metabolic routes in gene-edited A2780, CAL-51, and COV362 cells as well as in NIH3T3 cells ectopically expressing specific R-RAS2 mutants. As control, we used the appropriate parental cell lines. We observed that R-RAS2^Q72L^-deficient A2780 cells (Fig. 7C and Supplementary Fig. 5A) and R-RAS2^Q72L^-deficient CAL-51 cells (Fig. 7D and Supplementary Fig. 5B) had reduced levels of most mitochondrial metabolic parameters analyzed, including basal and maximal respiration; proton leak; ATP production; and spare respiratory capacity when compared to their respective controls (Fig. 7C, D). Interestingly, elimination of the endogenous wild-type R-RAS2 also impaired those mitochondrial parameters in COV362 cells (Fig. 7E and Supplementary Fig. 5C). This indicates that endogenous R-RAS2 is important for the regulation of key mitochondrial functions in all the cancer cell lines used in this study regardless of its mutational status. Therefore, it is unlikely that this change in mitochondrial activity could explain per se the reduced tumorigenic activity of RRAS2^Q72L^-deficient cells. Conversely, we observed that the ectopic expression of R-RAS2^Q72L^ and R-RAS2^G23V^, but not of wild-type R-RAS2, induced an increase in basal (Fig. 7F and Supplementary Fig. 5D) and maximal respiratory activity (Fig. 7G and Supplementary Fig. 5D); proton leakage (Fig. 7H and Supplementary Fig. 5D), and mitochondrial O_2_ consumption (Fig. 7I and Supplementary Fig. 5D) in NIH3T3 cells. This is a specific feature of the mutant *RRAS2* alleles, as we obtained similar data when analyzing the mouse fibrosarcoma FS#2 cell line that expresses R-Ras2^Q72L^ endogenously (Fig. 7J-M and Supplementary Fig. 5E). Collectively, these data indicate that the mutant versions of R-RAS2 contribute to the upregulation of respiratory activity in cells.

In contrast to the foregoing results, we found that the effect of endogenous and ectopically expressed R-RAS2 on glycolytic activity was highly cancer cell line-specific. Thus, in agreement with the microarray analyses, we found reduced glycolytic activity in *RRAS2*^Q72L^-knockout A2780 cells (Supplementary Fig. 5F). This defect was compensated when mitochondrial respiration was shut down (Supplementary Fig. 5G). By contrast, the activity of this metabolic pathway was upregulated in the case of *RRAS2*^Q72L^-deficient CAL-51 cells (Supplementary Fig. 5H). This feature was canceled out upon the shutdown of mitochondrial respiration in those cells (Supplementary Fig. 5I). In the case of COV362 cells, we also observed the upregulation of glycolytic activity in the absence of endogenous wild-type R-RAS2 (Supplementary Fig. 5J) although, unlike the case of CAL-51 cells, the lack of R-RAS2 led to severe defects in compensatory glycolytic activity in this case (Supplementary Fig. 5K). We could not find any significant effect of ectopically expressed R-RAS2^Q72L^ in glycolysis in NIH3T3 cells (Supplementary Fig. 5L), although the exhibited slightly higher levels of compensatory glycolysis than controls upon the shutdown of mitochondrial respiratory activity (Supplementary Fig. 5M). Collectively, these analyses indicate that, despite the different intrinsic metabolic features of each cell type, the expression of R-RAS2^Q72L^ usually correlates with increased mitochondrial respiratory activity in the cancer cells analyzed herein (Fig. 7N). Glycolysis, in contrast, is more dependent on the cell type (and probably the mutational package) of the interrogated cell lines used in this work.

## Discussion

Using a tamoxifen-inducible knock-in mouse model, our group recently demonstrated that the *RRAS2*^Q72L^ mutation acts as a bona fide oncogenic driver in vivo [18]. These results suggest that other gain-of-function mutations of *RRAS2* that are found at low frequency in human tumors and Noonan syndrome patients could also be oncogenic. Consistent with this idea, we have found that R-RAS2 proteins with mutations at residues Gly^23^, Gly^24^, Ala^70^ or Gln^72^ can transform NIH3T3 cells. Our signaling experiments also indicate that the oncogenic versions of R-RAS2 can potently activate the RAF–ERK and PI3K–AKT pathways in the transformed NIH3T3 cells. However, the output derived from the latter pathway seems to correlate more accurately with the oncogenic activity exhibited by the interrogated R-RAS2 mutants. Interestingly, we have found that some non-transforming mutants can specifically stimulate the RAF–ERK but not the PI3K–AKT pathway (D44E, E48D, K169Q, K159Q, A167T, R147Q). This suggests that these R-RAS2 mutants could contribute to oncogenic transformation as well if the cells that carry them acquire co-mutations that activate the latter signaling pathway. The results obtained with the oncogenic R-RAS2 mutants are to some extent expected, given that analogous mutations are also highly pathogenic in the context of RAS proteins (e.g., Gly^12^, Gly^13^, Gln^61^ and Ala^59^) [22]. Nonetheless, we also found that some non-transforming R-RAS2 mutants (e.g., D44E, P45R and A167T) are pathogenic in RAS proteins in cancer cells (D33E) or in RASopathies (P34R, F156L) [38–40]. This differential activity might be caused by a lack of proper activation of the PI3K–AKT pathway by these non-oncogenic R-RAS2 versions (Fig. 1D, E).

While results from ectopic expression systems might not adequately reflect the exact roles of the endogenous proteins, our in silico and wet lab analyses have demonstrated that the endogenous expression of R-RAS2^Q72L^ is required to maintain the full tumorigenicity of both ovarian and breast cancer cell lines. By contrast, the expression of the endogenous wild-type counterpart does not seem very relevant for this process, at least not for the cell line collection contained in the Novartis Drive Data Portal. It is worth noting, however, that this latter result could be cell type-specific, as we have previously shown that endogenous wild-type R-RAS2 is required for the in vivo tumorigenic properties of specific mouse and human breast cancer cell lines [23]. Likewise, using mouse models, we previously demonstrated that the expression of the endogenous wild-type R-Ras2 protein is quite relevant for Her2- or Notch1-mediated transformation of breast cancer or T cell acute lymphoblastic leukemia, respectively [18, 23]. Thus, it is likely that the requirements of the wild-type R-RAS2 protein depend on the mutational package or cell type of origin of the cancer cells. One of the most striking findings of these experiments is that the expression of endogenous R-RAS2^Q72L^ is required for cancer cells that harbor concurrent mutations in key signaling elements of the RAS route, such as BRAF, RAF1 and PI3Kα. These results suggest that R-RAS2 and RAS proteins likely cooperate to generate an optimal signaling output from these two signaling pathways. They also hint that mutations in *RRAS2* could promote resistance to RAS, MEK and PI3Kα inhibitors in *RAS* mutant-positive tumors.

Results from cell models using ectopically expressed proteins have led to the idea that R-RAS2 and RAS proteins are quite similar according to subcellular localization, transforming activity and signaling criteria [3–10, 19]. In contrast to these assumptions, we have recently reported that R-Ras2^Q72L^ and oncogenic versions of RAS proteins trigger different tumor types when expressed in mice [18, 41–45]. It is unlikely that this is merely due to distinct expression patterns, given that R-Ras2 can be detected in many tissues [2, 26]. Thus, we surmise that R-RAS2 and RAS proteins must have specific mechanistic features that cause different, tissue type-specific pathogenic programs. In line with this idea, our work has revealed that the endogenous wild-type and Q72L versions of R-RAS2 are specifically localized to focal adhesions. This localization is observed in cancer cell lines from different cancer types (ovary, breast, fibrosarcoma) and species (human and mouse), suggesting that it is likely a general feature of R-RAS2 proteins. Retrospectively, this localization in focal adhesions can explain why R-RAS2 has been detected in proteomics experiments in myosin II-responsive focal adhesion complexes [46]. It could also explain the cell spreading and adhesion induced by the ectopic expression of some Noonan syndrome-associated R-RAS2 mutants in HEK293T cells [19], although this effect could be associated with other unrelated mechanisms induced by the overexpressed proteins. We favor the latter explanation, as these R-RAS2 mutants were all localized on the plasma membrane and the Golgi apparatus rather than on focal adhesions in these cells [19]. The connection of endogenous R-RAS2 with focal adhesions seems to be functionally relevant, as we have seen that the elimination of endogenous R-RAS2^Q72L^ leads to fewer focal adhesions and alterations in cell adhesion, invasion and chemotactic responses in both A2780 and CAL-51 cells. These defects can be also influenced by the change in the spectrum of integrins and extracellular remodeling factors seen in *RRAS2*^Q72L^-knockout A2780 cells. In contrast, we did not find any overt defects in focal adhesion formation or related functions after elimination of endogenous wild-type R-RAS2 in COV362 cells. Further work in this area will be needed to fully understand the specific roles of the wild-type and oncogenic versions of R-RAS2 in this biological process. In any case, the results presented here indicate that R-RAS2 and RAS proteins are likely to engage common effectors using different subcellular platforms and upstream signals, a finding that might have important repercussions for our understanding of how each of those proteins work in the context of both cancer and Noonan syndrome. These data also reinforce the importance of addressing the role of the RAS superfamily of GTPases based on endogenous proteins rather than using ectopic expression systems. Interestingly, a localization in focal adhesions has been observed for a gain-of-function mutant version and, to a lesser extent the wild-type version of the related R-RAS GTPase in HeLa cells [47]. This subcellular localization was attributed to specific amino acid stretches located in the C-terminal tail of this R-RAS2-related protein. In addition, it required palmitoylation of the active GTP-bound version of the protein [47]. It is likely that a similar process is associated with the subcellular localization of R-RAS2, since its C-terminal tail has some resemblance to that found in this paralogue. Likewise, R-RAS2 has a potential palmitoylation site in that region. However, it should be noted that, unlike the case of R-RAS, we have found similar levels of localization in focal adhesions of wild-type and Q72L mutant versions of R-RAS2. More work should be done to narrow down all the structural and regulatory layers that contribute to the specific subcellular localization of R-RAS2.

Our results indicate that endogenous R-RAS2^Q72L^, but not wild-type R-RAS2, is also required for the proper engagement of a wide variety of general immune and inflammatory responses in cancer cells (TNFα, NFκB, IFN) that likely impact in both the innate and adaptive immune system cells. Endogenous R-RAS2^Q72L^ is also critical for maintaining proper mitochondrial respiratory and glycolytic activity by cancer cells. The connection of these metabolic pathways to the promotion of the in vivo tumorigenic fitness of cancer cells by this oncogenic protein remains unclear. Hence, in the case of mitochondrial respiratory activity, we found similar deleterious effects when the endogenous versions of oncogenic and wild-type R-RAS2 are knocked out in the cancer cells. However, the elimination of wild-type R-RAS2 does not impair the in vivo transforming activity of ovarian cancer cells. Moreover, in the case of glycolytic activity, we have found that the effects derived from the elimination or the overexpression of R-RAS2 are highly cancer cell type-dependent. Thus, the influence of R-RAS2 on in vivo tumorigenesis seems more likely to be associated with its effects in the regulation of optimal ERK and PI3Kα–AKT–mTORC signaling and, perhaps, in focal adhesion-related functions rather than due its impact on these two metabolic pathways.

Together with our recent work in mice [18], the present study reinforces the idea that the oncogenic *RRAS2* mutations found in tumors are likely important for both cancer initiation and maintenance. These results also reveal cancer-connected pathways and pathobiological programs that are heavily dependent on the expression of the mutant proteins. It will be important to assess in the near future whether some of these functions are also conserved in the tissues that are dysregulated in Noonan syndrome individuals who carry the *RRAS2* mutation.

## Materials and methods

### Mouse strains

The orthotopic transplantation experiments were performed using a *Vav1*^–/–^;*Vav2*^–/–^;*Vav3*^–/–^ immunocompromised mouse strain (C57BL/10 genetic background) described elsewhere [48, 49]. This lymphopenic mouse strain was used to minimize the potential rejection of the transplanted human cells [48]. Animals were kept in ventilated rooms in pathogen-free facilities under controlled temperature (23 °C), humidity (50%), and illumination (12-h-light/12-h-dark cycle) conditions. Before surgery, mice were injected with 0.1 mg buprenorphine/kg for pain relief and anesthetized by isofluorane inhalation (Vetflurane, cat. no. 575837-4, Virbac). All mouse experiments were performed according to protocols approved by the Bioethics Committee of the University of Salamanca and the animal experimentation authorities of the autonomous government of Castilla y León (Spain).

### Cell lines

All cell lines used in this work were authenticated and tested at the Salamanca University Hospital Service and regularly tested for *Mycoplasma* contamination using the MycoSEQ™ Mycoplasma detection kit (Thermo Fisher Scientific, Catalog No. 4460626). The cell lines NIH3T3 (cat. no. CRL-1658) and HEK293T (cat. no. CRL-1573) were obtained from the American Type Culture Collection. The ovarian cancer COV362 and COV504 cell lines were purchased from Sigma (cat. no. 07071910 and 07071902, respectively). The human breast carcinoma cell line CAL-51 was obtained from DSMZ (cat. no. ACC 302). All cells were grown in DMEM (Dulbecco’s Modified Eagle Medium) supplemented with 10% fetal bovine serum, 1% L-glutamine, penicillin (10 μg/ml) and streptomycin (100 μg/ml). The A2780 ovarian carcinoma cell line was provided by Dr. A. Pandiella (Cancer Research Center, Salamanca) (cat. no. 93112519, Sigma) and cultured in RPMI (Roswell Park Memorial Institute) media supplemented with 10% fetal bovine serum, 1% L-glutamine, penicillin (10 μg/ml) and streptomycin (100 μg/ml). Fibrosarcoma FS#2 cells isolated from R-Ras2^Q72L^-expressing mice are described elsewhere [18]. All cell lines were maintained at 37 °C in a humidified 5% CO_2_ atmosphere. In all cases, cells were dissociated using TripLE™ Express (cat. no. 12604013, GIBCO). In the case of cell stimulation, A2780 or COV362 cells were serum starved for 4 h and then stimulated with 20 ng/ml of either human TNFα (cat. no. 300-01A, Preprotech) or mouse IFNγ (cat. no. 315-05, Preprotech) for the indicated periods of time.

### Microbe strains

The *E. coli* DH5α strain was used for cloning and plasmid generation.

### Plasmids

Vectors encoding the different R-RAS2 mutants were generated by site-directed mutagenesis. pBlueScript SK(+)-HA-R-RAS2^WT^ was used as a template for the site-directed mutagenesis using the primers listed in Supplementary Table 4. The mutant pBlueScript SK(+)-HA-R-RAS2 plasmids were subsequently BamHI-digested and inserted into the mammalian expression vector pEF1/V5-HisA.

### Focus formation assays

NIH3T3 cells were seeded at a density of 1.25 × 10^5^ cells/ml in six-well plates and transfected with 1 µg of the appropriate vectors using the Lipofectamine™ 2000 Transfection Reagent (cat. no. 11668019, ThermoFisher Scientific). After 24 h, transfected cells were split into two 100-mm plates and maintained for 11 to 14 days, with media changes every 3 days, until colonies were visible. Cells were then fixed with 4% formaldehyde (cat. no. 252931, PanReac AppliChem) for 10 min and stained with Giemsa (1:10, cat. no. 32884, Merck) for 10 min. Plates were washed with distilled water and left to dry, and colonies were counted.

### Ectopic expression of R-RAS2 mutants in mouse fibroblasts

pEF1/V5-HisA-HA-R-RAS2–derived plasmids were used to ectopically express the indicated HA-tagged versions of R-RAS2 in NIH3T3 cells. To this end, NIH3T3 cells were transfected with 1 µg of the different plasmids using Lipofectamine™ 2000 Transfection Reagent. Selection with 800 μg/ml of G418 led to the establishment of stable cell lines overexpressing the different HA-R-RAS2 proteins.

### Western blotting

For total protein analyses, cells were washed in phosphate-buffered saline solution twice and lysed in RIPA buffer (10 mM Tris-HCl [pH 7.5], 150 mM NaCl, 1% Triton X-100, 1 mM NaF, 1 mM Na_3_VO_4_, and a cOmplete™ protease inhibitor cocktail tablet [cat. no. 05056489001, Roche]). In the case of analysis of phosphorylated proteins, cells were washed as above and lysed in BLYS buffer (50 mM Tris-HCl [pH 7.5], 1 mM EGTA, 1 mM EDTA, 1% Triton X-100, 50 mM NaF, 1 mM Na_3_VO_4_, 5 mM Na_4_P_2_O_7_, 0.27 M sucrose, 1 mM phenylmethylsulfonyl fluoride and a cOmplete™ protease inhibitor cocktail tablet). In all cases, cellular extracts were precleared by centrifugation at 20,000 × *g* for 10 min at 4 °C, denatured by boiling in 5× SDS-PAGE sample buffer, separated electrophoretically, and transferred onto nitrocellulose filters (cat. no. 2022-04-26, ThermoFisher Scientific) using the iBlot Dry Blotting System (cat. no. IB21001, ThermoFisher Scientific). Membranes were blocked in 5% bovine serum albumin (cat. no. A4503, Sigma-Aldrich) in TBS-T (25 mM Tris-HCl [pH 8.0], 150 mM NaCl, 0.1% Tween-20) for at least 1 h and then incubated overnight at 4 °C with the appropriate antibody. Primary antibodies used included those directed to R-RAS2 (made in-house; 1:1000 dilution; see below for description), tubulin α (cat. no. CP06-100UG, Calbiochem, 1:2000 dilution), phospho-ERK1/2 (p-Thr^202^ and p-Tyr^204^, cat. no. 4370, Cell Signaling Technology, 1:1000 dilution), total ERK1/2 (cat. no. 9102, Cell Signaling Technology, 1:1000 dilution), phospho-AKT (p-Thr^308^, cat. no. 4056, Cell Signaling Technology, 1:1000 dilution), phospho-AKT (p-Ser^473^, cat. no. 4051, Cell Signaling Technology, 1:1000 dilution), total AKT (cat. no. 2920, Cell Signaling Technology, 1:1000 dilution), phospho-MEK1^S298^ (cat. no. 98195, Cell Signaling Technology, 1:1000 dilution), total MEK1 (cat. no. 2352, Cell Signaling Technology, 1:1000 dilution), phospho-NF-kB^S536^ (cat. no. 3033. Cell Signaling Technology, 1:1000 dilution) and NF-kB p65 (cat. no. 8242. Cell Signaling Technology, 1:1000 dilution). Membranes were then washed three times with TBS-T, incubated with the appropriate secondary antibody (ECL Rabbit IgG, HRP-linked whole Ab, cat. no. NA934-1ML, Amersham, 1:5000 dilution; ECL Mouse IgG, HRP-linked whole Ab, cat. no. NXA931-1ML, Amersham, 1:5000 dilution) for 60 min at room temperature and washed again three times as above. Immunoreactive bands were visualized using a chemiluminescent method (ECL Pierce, cat. no. 32106, ThermoFisher Scientific). Bands were quantified using the ImageJ software, using the tubulin signal as a normalization control for samples.

### Heatmaps for signaling experiments

Heatmaps were generated using the heatmap3 *R* package (http://CRAN.R-project.org/package=heatmap3).

### In silico gene dependency determinations

Data for indicated genes were generated using the Drive Data Portal (https://oncologynibr.shinyapps.io/drive/).

### CRISPR–Cas9 gene editing

CRISPR–Cas9 was used to knock out the *RRAS2* gene in A2780, COV362 or COV504 cells, based on the non-homologous end joining repair pathway. The plasmids used contain the Cas9-2A–GFP and a guide RNA (sgRNA, plasmids pLCM71-73) (Supplementary Table 5). Synthetic sgRNAs targeting the *RRAS2* gene (Supplementary Table 5) were designed using open access online tools, such as www.crispr.mit.edu and the Benchling CRISPR design tool (www.benchling.com). Upon phosphorylation and annealing, the DNA oligos containing the sequences for the sgRNAs were cloned (using BbsI sites) into the pSpCas9(BB)-2A-GFP (PX458) vector (cat. no. 48138, Addgene), to generate the pLCM71-73 plasmids. Cells exponentially growing in 6-cm plates were transfected using Lipofectamine™ 2000 Transfection Reagent with 2.5 µg plasmid containing the Cas9 and the sgRNAs. GFP-positive cells were isolated after 36 h by flow cytometry using a BD FACSAria III cell sorter in 96-well plates. Cells were grown and maintained as independent clones. Proper knockout was assessed using DNA sequencing and immunoblot analyses using antibodies to R-RAS2.

To knockout the *RRAS2* gene in CAL-51 cells, a CRISPR–Cas9 approach based on the homology-directed repair (HDR) pathway was used. To this end, two plasmids were generated, one encoding the Cas9 and the sgRNA (plasmids pLCM74-76) and the other containing the donor DNA for the HDR step (pLCM79) (Supplementary Table 5). Synthetic sgRNAs targeting the 25 bp surrounding the ATG position of the *RRAS2* gene (Supplementary Table 5) were designed using the Benchling CRISPR design tool. After phosphorylation and annealing, the DNA oligos containing the sequences for the sgRNAs were cloned into the BbsI-digested pX330-U6-Chimeric_BB-CBh-hSpCas9 vector (cat. No. 42230, Addgene), to generate the pLCM74-76 plasmids. For the second plasmid, a pBlueScript SK ii (+) vector (cat. no. 212205, Addgene) containing the EGFP sequence was used as backbone vector (pJG1). Two DNA fragments homologous to the genomic region upstream (left homology arm, LHA) or downstream (right homology arm, RHA) of the ATG of the *RRAS2* gene were PCR-amplified using genomic DNA from A2780 cells (Supplementary Table 5). To introduce the homology fragments into the backbone vector, the primers used to amplify the genomic DNA were extended with overhang sequences containing a HindIII/BamHI (for the LHA) and XhoI/NheI (for the RHA) sites. The LHA and RHA were sequentially cloned into the backbone vector, giving rise to an insertion of the GFP-polyA and a subsequent frameshift in the *RRAS2* sequence (pLCM79 plasmid). Cells growing exponentially in 6-cm plates were transfected using Lipofectamine 2000 with 1.0, 1.5, 2.0 or 3.0 μg of total DNA with a 1:2 pLCM74-76:pLCM79 ratio. After 24 h, cells were again transfected with 1.0, 1.5, 2.0 or 3.0 μg of pLCM79 alone. At 36 h after the second transfection, EGFP-positive cells were isolated by flow cytometry using a BD FACSAria III cell sorter. Western blots and PCR analyses using genomic DNA were used to screen for the proper generation of *RRAS2* knockout independent clones.

For EGFP tagging of human and mouse proteins, a CRISPR–Cas9 approach based on the HDR pathway was used. To knock-in the EGFP tag in human cell lines, the pLCM74-76 plasmids (encoding Cas9 and the sgRNAs; see above) and the pLCM77 plasmid (containing the donor DNA for the HDR step) were used (Supplementary Table 5). The pLCM77 plasmid was generated in a similar way as described for pLCM79 (see above), except that the EGFP-encoding sequence was inserted in-frame with the *RRAS2* sequence. For mouse cells, the pMFP23-24 (encoding Cas9 and the sgRNA) and the pMFP29 (containing the donor DNA for the HDR step) plasmids were used (Supplementary Table 5). However, in this case, the PAM sequence in the donor DNA was mutated using the primers mPAM2 mut F and mPAM2 mut R (Supplementary Table 5). In all cases, cells were transfected, enriched, subcloned and characterized as above.

### Generation of antibodies to R-RAS2

This polyclonal antibody has been described elsewhere [18]. Briefly, a synthetic peptide corresponding to the R-RAS2 C-terminus (CPPSPEPTRKEKDKKG) was used to immunize rabbits using the Invitrogen service. Antibodies were purified using an affinity column coated with the immunizing peptide. Antibodies were tittered by enzyme-linked immunosorbent assays, and their specificity was tested by Western blot using control and R-RAS2 knockout cells. Experiments showed that this antibody recognizes R-RAS2 but not R-RAS or the RAS proteins.

### Proliferation assays

To assess proliferation using the 3-(4,5dimethylthiazol-2-yl) 2,5-diphenyltetrazolium bromide (MTT) method, cells were seeded in 24-well plates at either 3 × 10^4^ (A2780 and COV362) or 4 × 10^4^ (COV504) cells/well. At the indicated time points, the culture medium was replaced by 250 μl of the MTT solution (0.5 mg/ml); after 1 h at 37 °C in a 5% CO_2_ atmosphere, the MTT solution was replaced by 500 μl dimethyl sulfoxide to dissolve the formazan crystals that had formed. After 10 min, the absorbance at 570 nm was measured in a Tecan Ultra Evolution plate reader. For CAL-51 cells, cells were plated onto 24- or 6-well plates at a density 4.5 × 10^4^ cells/well. At the indicated times, cells were collected, diluted 1:10 in Beckman Counter ISOTON II (cat. no. 8448011, Beckman), and counted using the Beckman Coulter Z1 apparatus.

### Orthotopic xenografts in mice

For the orthotropic transplant of ovarian cancer cell lines, the left ovaries of 8-to-10-week-old female *Vav1*^–/–^;*Vav2*^–/–^;*Vav3*^–/–^ mice were exposed by a dorsal incision and intrabursally injected with 1 × 10^6^ cells (in 6 μl) using a 30-gauge beveled glass needle. For this step, exponential cultures of A2780 cells were trypsinized, resuspended in complete medium, counted, pelleted by centrifugation, and resuspended in phosphate-buffered saline solution supplemented with colorant solution fast green (cat. no. F7258, Sigma). Following the intrabursal injection, abdominal muscles and skin of the mice were closed with surgical suture. Tumors were collected and weighted 2 weeks later.

In the case of in vivo experiments with breast cancer cells, they were trypsinized, resuspended in complete medium, counted, pelleted by centrifugation, washed once with ice-cold phosphate-buffered saline solution, re-pelleted, resuspended in a 1:1 solution of growth factor reduced and phenol red-free Matrigel (cat. no. 356231, BD Biosciences):phosphate-buffered saline solution to a concentration of 5 × 0^7^ cells/ml. One hundred microliters of this cell suspension were then subcutaneously injected into the right inguinal mammary gland of anesthetized, age-matched 4- to 16-week-old female *Vav1*^–/–^;*Vav2*^–/–^;*Vav3*^–/–^ mice using a 25-gauge needle. Tumor growth was monitored weekly for 4 weeks. Upon euthanasia, the tumors were isolated and weighted.

### Immunofluorescence experiments

Cells were cultivated at the desired density and grown over night on cover slides pre-treated with 2 µg/ml fibronectin in imaging buffer (20 mM HEPES [pH 7.4], 140 mM NaCl, 2.5 mM KCl, 1.8 mM CaCl_2_ and 1 mM MgCl_2_) for 1 h at 37 °C. Cells were then fixed in 4% formaldehyde in phosphate-buffered saline solution for 10 min at room temperature. After washing twice with phosphate-buffered saline solution, cells were permeabilized with 0.3% Triton X-100 (cat. no. X100, Sigma) in phosphate-buffered saline for 10 min at room temperature, washed three times with phosphate-buffered saline, blocked in 2% bovine serum albumin in TBS-T (25 mM Tris– HCl [pH 8.0], 150 mM NaCl, 0.1 % Tween-20) for 30 to 45 min in a wet chamber, incubated for 2 h at room temperature with antibodies to vinculin (cat. no. V9131, Sigma, diluted 1:5000 in blocking buffer), washed thrice in TBS-T, incubated with goat antibodies to mouse IgG coupled to Alexa Fluor 594 (cat. no. A11032, Invitrogen, dilution 1:400) for 30 min at room temperature and, when indicated, stained with Alexa Fluor 365-labeled phalloidin (cat. no. A34054, dilution 1:400) for 30 min at room temperature. After washing twice with TBS-T, cells were finally stained with 1 μg/ml of 4′,6-diamidino-2-phenylindole (cat. no. D1306, Invitrogen) for 3 min to visualize the nuclei. Images were captured in a Laser Scan Confocal Microscopy Leica SP8.

### Cell adhesion

Cells (4 × 10^4^ for A2780 cells, and 3 × 10^4^ for CAL-51 or COV362 cells) were seeded onto a P24 plate and, at the indicated time points, were washed and trypsinized using TripLE™ Express (cat. no. 12604013, GIBCO). Resuspended cells were diluted 1:20 in the Beckman Coulter ISOTON II buffer and counted in a Beckman coulter Z1 Coulter Particle Counter. Cell numbers at the endpoint were used to normalize the measurements.

### 3D cell migration and invasion

Microfluidic chips were fabricated in polydimethylsiloxane (Sylgard 184, Dow Corning) at a 10:1 ratio of base to curing agent, using SU8-silicon wafers obtained by soft lithography as indicated [50]. Polydimethylsiloxane microdevices were individualized and sterilized by wet and dry cycles of autoclaving. Surface plasma treatment was used to bond the polydimethylsiloxane with the glass-bottom of 35-mm Petri dishes (cat. no. 81156, Ibidi) followed by the addition of poly-D-lysine solution (cat. no. A-003-M, Sigma-Aldrich) at 1 mg/ml to enhance the surface–matrix attachment. The geometry of the microfluidic devices consisted of a central channel, in which the hydrogels were introduced and two media channels at both sides of the central channel that allowed for the introduction of cell culture medium. Further details about the geometry of the microfluidic devices can be found elsewhere [51]. For the cell migration experiments, A2780 and COV362 cells were mixed with hydrogel solutions at a final concentration of 2 × 10^5^ cells/ml, using collagen type I hydrogel (cat. no. 40236, BD Biosciences) that had been prepared to a final concentration of 2.5 mg/ml with Dulbecco’s phosphate-buffered saline (cat. no. BE17-512Q, Lonza), as indicated [50]. The hydrogel dilution was then pipetted into the central gel chamber and allow to be confined by surface tension. Once in place, the collagen gel solution was polymerized in a humidified chamber at 37 °C and 5% CO_2_ for 20 min. The hydrogel was then hydrated with DMEM supplemented with 10% fetal calf serum and incubated overnight for stabilization of matrix and cell adhesion. In the case of gradients with insulin growth factor 1 (cat no. PHG0071, ThermoFisher Scientific), the ligand (20 ng/ml in culture medium) was added to only one channel while new medium was added to the other channel without ligand. The chemical gradient was established by a diffusive process across the hydrogel [51, 52]. Time-lapse imaging was carried out with a Nikon D-Eclipse Ti Microscope with a 10× objective, acquiring phase contrast images every 20 min for 24 h. The focal plane was selected to be in the middle along the z-axis of the device to ensure that the tracked cells were embedded within the 3D network. During the experiment, the incubation conditions were controlled and held at 37 °C, 5% CO_2_, and 95% of humidity. Cell tracking was performed using a hand-coded semi-automatic MATLAB script as described previously [53]. Acquired data were then analyzed in terms of mean and effective speeds. By comparing pixel intensities and using matrix convolution techniques, the software was able to find and track cell centroids, request visual correction from the user, and post-process the migration results. These trajectories were used to extract the cell mean (*V*_mean_) and effective (*V*_eff_) velocities. Note that *V*_mean_ is defined as the averaged instantaneous speed including all time steps, whereas *V*_eff_ considers only the initial and final positions.

### Gene expression profiling and bioinformatic analyses

Total RNAs from control and *RRAS2* knockout A2789 cells were isolated using the RNAeasy Mini Kit (cat. no. 74104, Qiagen) and analyzed using Affymetrix microarrays (Clariom™ S Assay HT) at the CIC Genomics Core Facility according to the manufacture’s recommendations. Microarray data were bioinformatically analysed exactly as indicated in a previous publication [18]. Briefly, we used the *R* version 3.6.3 (https://stat.ethz.ch/pipermail/r-announce/2020/000650.html) and the Python version 3.9.0 (https://www.python.org/downloads/release/python-390/) for statistical analyses and text file processing, respectively. Signal intensity values were obtained from expression microarray CEL files after robust multichip average (http://bioconductor.org/packages/release/bioc/html/affy.html). Differentially expressed genes were identified using linear models for microarray data (limma, https://bioconductor.org/packages/release/bioc/html/limma.html). Adjusted *p*-values for multiple comparisons were calculated applying the Benjamini-Hochberg correction (FDR). Gene Ontology and KEGG pathways enrichment analyses were performed using the DAVID online platform (https://david.ncifcrf.gov). Expression heatmaps were generated using the heatmap3 *R* package (http://CRAN.R-project.org/package=corrplot). Volcano plots were generated using the Glimma *R* package (https://bioconductor.org/packages/release/bioc/html/Glimma.html). GSEA were performed with described gene sets using gene set permutations (*n* = 1000) for the assessment of significance and signal-to-noise metric for ranking genes (http://software.broadinstitute.org/gsea/index.jsp). The gene sets used were those from the Molecular Signatures (MSigDB v7.2; https://software.broadinstitute.org/cancer/software/gsea/wiki/index.php/MSigDB_v7.2_Release_Notes) and the Harmonizome (https://maayanlab.cloud/Harmonizome/) databases.

### Determination of mRNA levels

Total RNAs were extracted using the RNeasy® Mini Kit (Cat. # 74104, Qiagen) and qRT-PCR performed using the Power SYBR Green RNA-to-CT 1-Step Kit (Cat. # 4389986, Applied Biosystems) and the StepOnePlus Real-Time PCR System (Cat. # 4376600, Applied BioSystems). Raw data were analyzed using the StepOne software (Applied Biosystems). The abundance of the endogenous *GAPDH* mRNA was used as an internal normalization control. Primers used for transcript quantitation included 5′-GAG CTG TGA CTA CTT TGC CG-3′ (forward) and 5′-GAA AGG AAA CCA CGT CGC TT-3′ (reverse) for *ITGA5*; 5′-AGA CGT TTG GGA GAT TCG GT-3′ (forward) and 5′-GAA CTT GGG AAG GCT TGG TG-3′ (reverse) for *ITGA8*; 5′-AGA CGC GGG ATA TGA TTG GT-3′ (forward) and 5′-AGG TAG TGG CTA TCA GGG GA-3′ (reverse) for *ITAG7*; 5′-TTC TGT AGC TGC CAC TGA CA-3′ (forward) and 5′-CAA ACC GTG CAA AGA CCT CA-3′ (reverse) for *ITGAV*; 5′-GAG GTG TCT CAA GTG CCT GTAC-3′ (forward) and 5′-ACT GGC AGT CAG AGC CGT TAC A-3′ (reverse) for *LAMB1*; 5′-CTG TTC ATG CCA AGA GCC TGG T-3′ (forward) and 5′-TTT GCC GAG ACT GGA GGA TTC C-3′(reverse) for *PARVA*; 5′-GTC TTT GAC TCC TTG CTG AAT CTG-3′ (forward) and 5′-CAC CTC ATC GTC TTC CAA GCAC-3′ (reverse) for *CLDN1*; 5′-GTG GAA GGT GAC CGC TTT CAT C-3′ (forward) and 5′-CAG CAG TGA GTC GTA CAC CTT G-3′ (reverse) for *CLDN6*; 5′-GGC TTC ATA GCA TTC GCC TAC TC-3′ (forward) and 5′-AGA TGT TCA GGC ACT TGG CGG T-3′ (reverse) for *IFITM1*; 5′-GGC TTC ATA GCA TTC GCG TAC TC-3′ (forward) and 5′-AGA TGT TCA GGC ACT TGG CG GT-3′ (reverse) for IFITM2; 5′-CTG GGC TTC ATA GCA TTC GCC T-3′ (forward) and 5′-AGA TGT TCA GGC ACT TGG CGG T-3′ (reverse) for *IFITM3*; 5′-GAT GAA GCA GCC CAG ATG TG-3′ (forward) and 5′-GCT TGA CCC TCA GAG ACC TT-3′ (reverse) for *MMP1*; 5′-CCT GGA AAT GTT TTG GCC CA-3′ (forward) and 5′-TCA TCT TGA GAC AGG CGG AA-3′ (reverse) for *MMP3*; 5′-CTG CCT ATC CTC TGA GTG GG-3′ (forward) and 5′-CAC CTC CAG AGT GTC AGT GT-3′ (reverse) for *MMP10*; 5′-TTG GAC CTG GAT CTG GCA TT-3′ (forward) and 5′-GCC ACG TAT GTC ATC AGC AG-3′ (reverse) for *MMP12*; 5′-AGA AGG CTC CAG CCA TCT CTG T-3′ (forward) and 5′-TGC TGG TAG AGT TCG GTG CAG A-3′ (reverse) for *IFNA*; 5′-GAG TGT GGA GAC CAT CAA GGA AG-3′ (forward) and 5′-TGC TTT GCG TTG GAC ATT CAA GTC-3′ (reverse) for *IFNG*; 5′-AGT GCT TAG CCT GGT ATT CAT CTG-3′ (forward) and 5′-GGC TGG TAT GAC GTG ATG AGT G-3′ (reverse) for *IFNGR1*; 5′-CTC CAT TCT GCC TGG GTG ACA A-3′ (forward) and 5′-CGT GGA GGT ATC AGC GAT GTC A-3′ (reverse) for *IFNGR2*; and 5′-GAG TCA ACG GAT TTG GTC GT-3′ (forward) plus 5′-TTG ATT TTG GAG GGA TCT CG-3′ (reverse) for *GAPDH*.

### Flow cytometry

Exponentially growing A2780 and A2780 KO#C20 cells were detached using non-enzymatic dissociation reagent (cat. no. 13151014, Thermo Fisher Scientific) for 10 min at 37 °C to prevent enzymatic-dependent integrin shedding. Cells were then centrifuged in 10 ml of phosphate-buffered saline solution containing 1% bovine serum albumin and 2 mM EDTA (PBS* solution) and transferred onto ice to be incubated with antibodies to integrin antibodies (1:200 dilution in PBS*) (FITC-labeled to human integrin α5, CD49e, clone NKI-SAM-1 [cat. no. 328007, Biolegend]; Alexa Fluor® 647-labeled to human integrin β1, CD29, clone TS2/16, [cat. no. 303017, Biolegend] for 40 min at 4 °C. After rinsing with 2 ml of ice-cold PBS*, cells were resuspended in 200 μl of PBS* and analyzed in a FACSAria® III flow cytometer (Becton Dickinson). For representation, we normalized the fluorescence intensity on the FITC (integrin α5) channel with the intensity obtained in the channel for the AlexaFluor647®-labeled antibody to integrin β1, which remained unchanged. We performed two independent experiments with technical quadruplicates for each experimental point.

### Metabolic analyses

Oxygen consumption (OCR) and extracellular acidification (ECAR) rates were measured in a Seahorse XFe24 Analyzer (Agilent). For this, 40,000 cells/well (for A2780, COV362, FS#2 or NIH3T3 cells) or 60 000 cells/well (for CAL-51 cells) were seeded and grown overnight in pre-treated Seahorse XF24 V7 PS Cell Culture Microplates (cat. no. 102342-100, Agilent). Such pre-treatment was carried out using either cell-tak (cat. no. 10317081, Corning) at 22.4 μg/ml for 20 min at room temperature (for NIH3T3 and FS#2 cells) or fibronectin (2 μg/ml; cat. no. F1141-1MG, Sigma) for 4 h at 37 °C (for A2780, CAL-51 and COV362 cells). Both cell-tak and fibronectin were diluted in imaging buffer. Prior to the assay, cells were washed and incubated for 1 h at 37 °C in a non-CO_2_ incubator with Seahorse phenol red-free DMEM (cat. no. 103575-100) supplemented with 1 mM pyruvate (cat. no. 103578-100, Agilent), 2 mM glutamine (cat. no. 103579-100, Agilent), and 10 mM glucose (cat. no. 103577-100, Agilent). In the Mito Stress test, 1.5 μM oligomycin (cat. no. 495455, Millipore), 1 μM carbonyl cyanide-p-trifluoromethoxy phenyl-hydrazone (FCCP; cat. no. C2920, Sigma), and a mix of 1 μM antimycin A (cat. no. A8674-50MG, Sigma) plus 1 μM rotenone (cat. no. R8875-1G, Sigma) were sequentially added to the cells. For glycolytic rate assays, the mix of 1 μM antimycin A and 1 μM rotenone was used in the first injection, followed by 50 mM 2-deoxy-D-glucose (cat. no. D6134-1G, Sigma). Compounds were diluted in the assay media and loaded into an overnight-hydrated XFe24 sensor cartridge (cat. no. 102342-100, Agilent) using the Seahorse CF calibrant solution (cat. no. 102342-100, Agilent).

### Statistics

Statistical analyses were carried out using GraphPad Prism software (version 6.0 and 8.0). The number of biological replicates (*n*), the type of statistical tests performed, and the statistical significance for each experiment are indicated in the figure legends. Data normality was tested using the Shapiro-Wilk test. Parametric distributions were analyzed using Student’s *t*-test (for comparing two experimental groups) or one-way ANOVA followed by either Dunnett’s test (for comparing more than two experimental groups with a single control group) or Tukey’s honestly significant difference (HSD) test (for comparing more than two experimental groups with every other group). Nonparametric distributions were analyzed using Mann-Whitney (for comparisons of two experimental groups). To analyze two factors, 2-way ANOVA was used, followed by either Tukey’s HSD test (for comparing more than two experimental groups with every other group) or Sidak’s test (for several independent comparisons).

## Supplementary information

Supplemental Information

Supplementary Table 1

Supplementary Table 2

Supplementary Table 3

Raw Data

## Data Availability

Microarray data reported in this paper have been deposited in the GEO database (https://www.ncbi.nlm.nih.gov/geo/) under the accession number GSE201472.

## Materials availability

All relevant data are available from the corresponding author upon reasonable request. A Materials Transfer Agreement could be required in the case of potential commercial applications.
