# Supplementary material for: Characterization of mutant versions of the R-RAS2/TC21 GTPase found in tumors
Source: Oncogene. 2022 Dec 7;42(5):389–405. doi: 10.1038/s41388-022-02563-9 (PMC9883167; doi:10.1038/s41388-022-02563-9)
Supplement: Supplementary file 1 — Supplemental Information [file 41388_2022_2563_MOESM1_ESM.pdf]

Supplementary Information for

Characterization of mutant versions of the R-RAS2/TC21 GTPase found in tumors

by

Laura Clavaín, Isabel Fernández-Pisonero *et al*.

This PDF file includes:

Supplementary Figures 1 to 5 and legends

Supplementary Tables 4 and 5

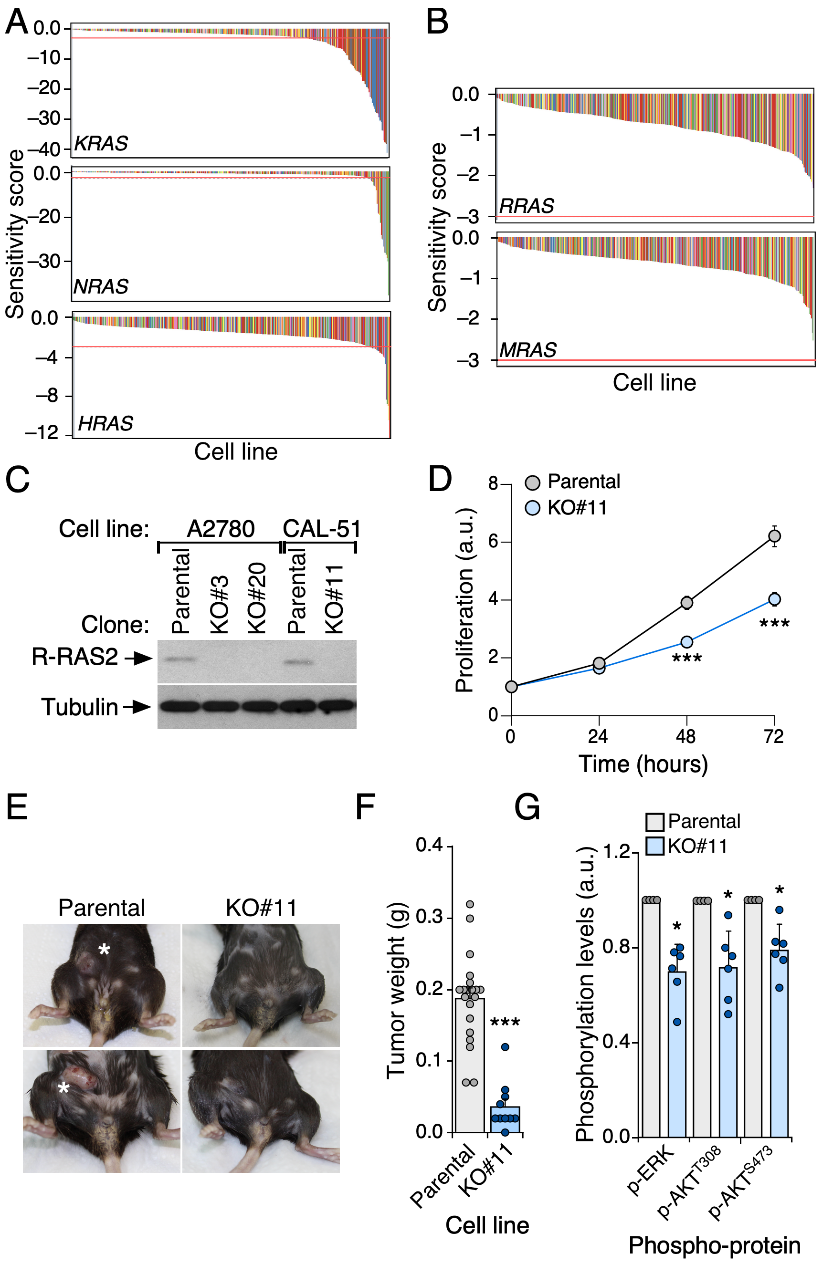

SUPPLEMENTARY FIGURE 1. Endogenous R-RAS2^{Q72L} is required for tumorigenesis of cancer cells

(A and B) Plot illustrating the dependency of cancer cell lines on indicated RAS (A) and RRAS (B) subfamily GTPases. The minimal sensitivity score established in each case is indicated by a horizontal red lane.

(C) Representative immunoblot analysis showing the levels of expression of indicated proteins (left) in lysates from gene-edited A2780 and CAL-51 cells (top). Levels of tubulin α were used as a loading control ($n = 3$).

(D) Proliferation of parental and *RRAS2*^{Q72L} knockout CAL-51 cells. Data represent the mean ± SEM. ***, $p < 0.001$ using two-way ANOVA and Tukey's multiple comparison test ($n = 3$).

(E and F) Representative images (E) and weight (F) of tumors formed by indicated CAL-51 cells upon implantation in the mammary pad of recipient animals. In E, asterisks indicate the tumor formed. In F, bars represent the mean ± SEM. ***, $p < 0.001$ using student's t-test. $n = 20$ and 10 for parental and CAL-51 KO#11 cells, respectively.

(G) Phosphorylation levels of indicated signaling proteins (bottom) in parental and *RRAS2*^{Q72L} knockout CAL-51 cells. Points represent independent experiments. Bars represent the mean ± SEM. *, $p < 0.05$ using Mann-Whitney test (n = 3).

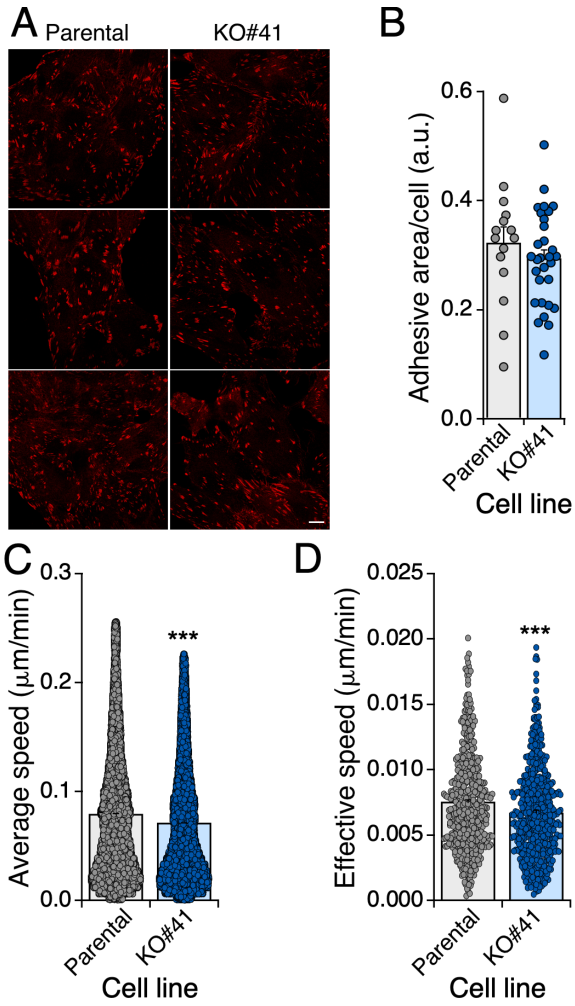

SUPPLEMENTARY FIGURE 2. Endogenous R-RAS2^{Q72L} regulates cell adhesion and migration

(A) Representative confocal microscopy images of indicated COV362 cells (top) upon staining with antibodies to vinculin. Scale bar, 10 µm.

(B) Quantitation of the total area of focal adhesions per cell from the experiments shown in A. Points represent individual cells. Bars represent the mean ± SEM. Data were statistically analyzed using Mann-Whitney test ($n = 3$).

(C and D) Average (C) and effective (D) speed in collagen-based hydrogels of indicated CAL-51 cells. Points represent individual cells. Bars represent the mean ± SEM. ***, $p < 0.001$ using Mann-Whitney test ($n = 3$).

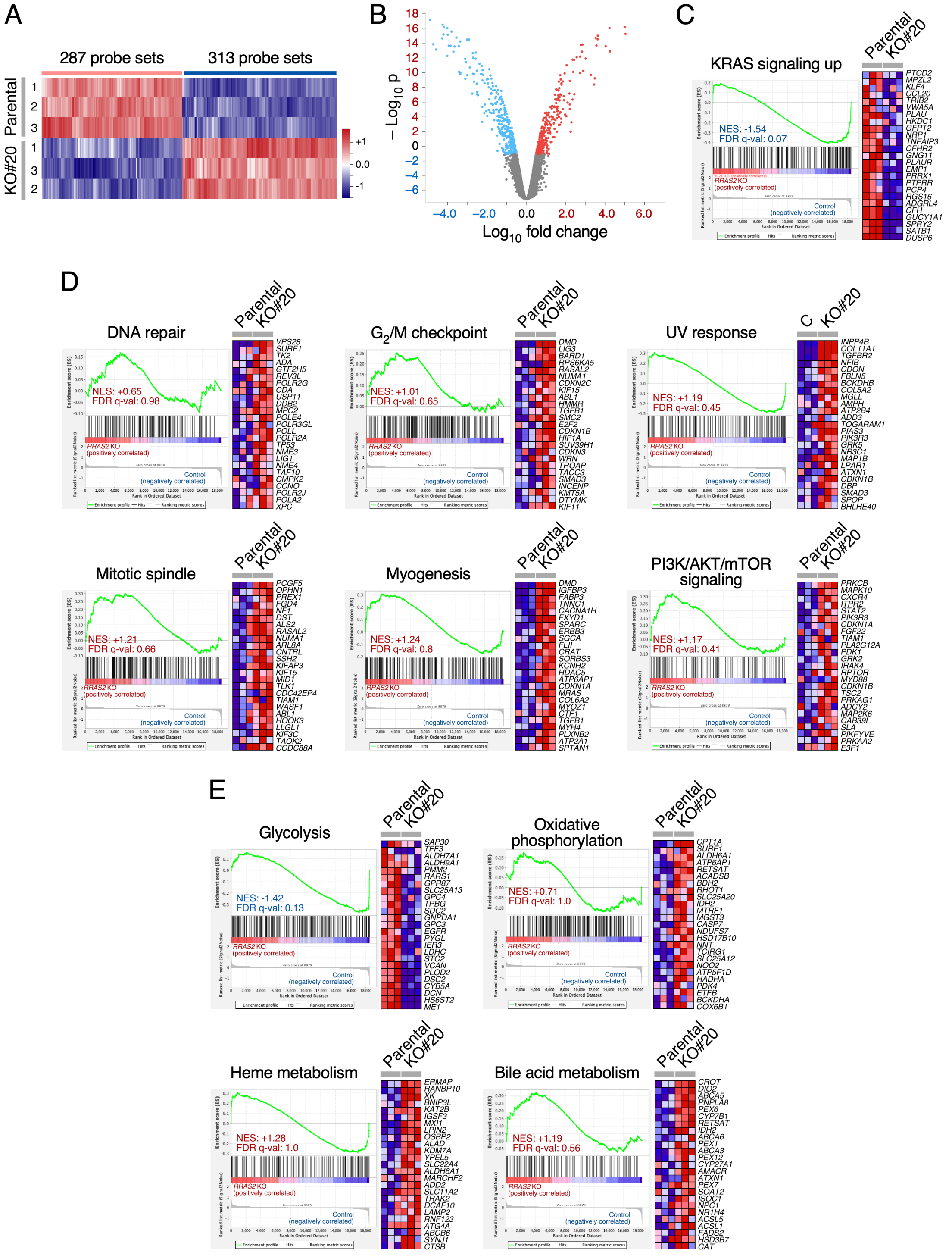

SUPPLEMENTARY FIGURE 3. Impact on the transcriptome of endogenous R-RAS2^{Q72L}

(A) Heatmap of transcripts up- (red) and downregulated (blue) in *RRAS2*^{Q72L} knockout (KO#20) versus control (Parental) A2780 cells. Rows represent independent replicates. Total number of transcripts is indicated at the top. The level of expression is shown in a gradient from dark blue (lowest) to dark red (highest) as indicated in the scale on the right.

(B) Volcano plot of transcripts up- (red) and downregulated (blue) in *RRAS2*^{Q72L} knockout (KO#20) versus control (parental) A2780 cells according to data generated in A. Transcripts that show no statistically significant variations over the established fold-change threshold are shown in grey.

(C) *KRAS* oncogene-related gene signature that is preferentially enriched in the downregulated transcriptome of *RRAS2*^{Q72L} knockout A2780 cells. The FDR q value and NES (normalized enrichment score) for the downregulated gene signatures are indicated inside the plot.

(D and **E)** Examples of gene signatures enriched in the transcriptome of *RRAS2*^{Q72L} knockout A2780 cells. The FDR q value and NES (normalized enrichment score) for the downregulated gene signatures are indicated in each plot using either blue (gene signatures enriched in the downregulated transcriptome; panel E, glycolysis) or red (gene signatures positively enriched in the upregulated transcriptome, panel D and E, rest of signatures) or color fonts.

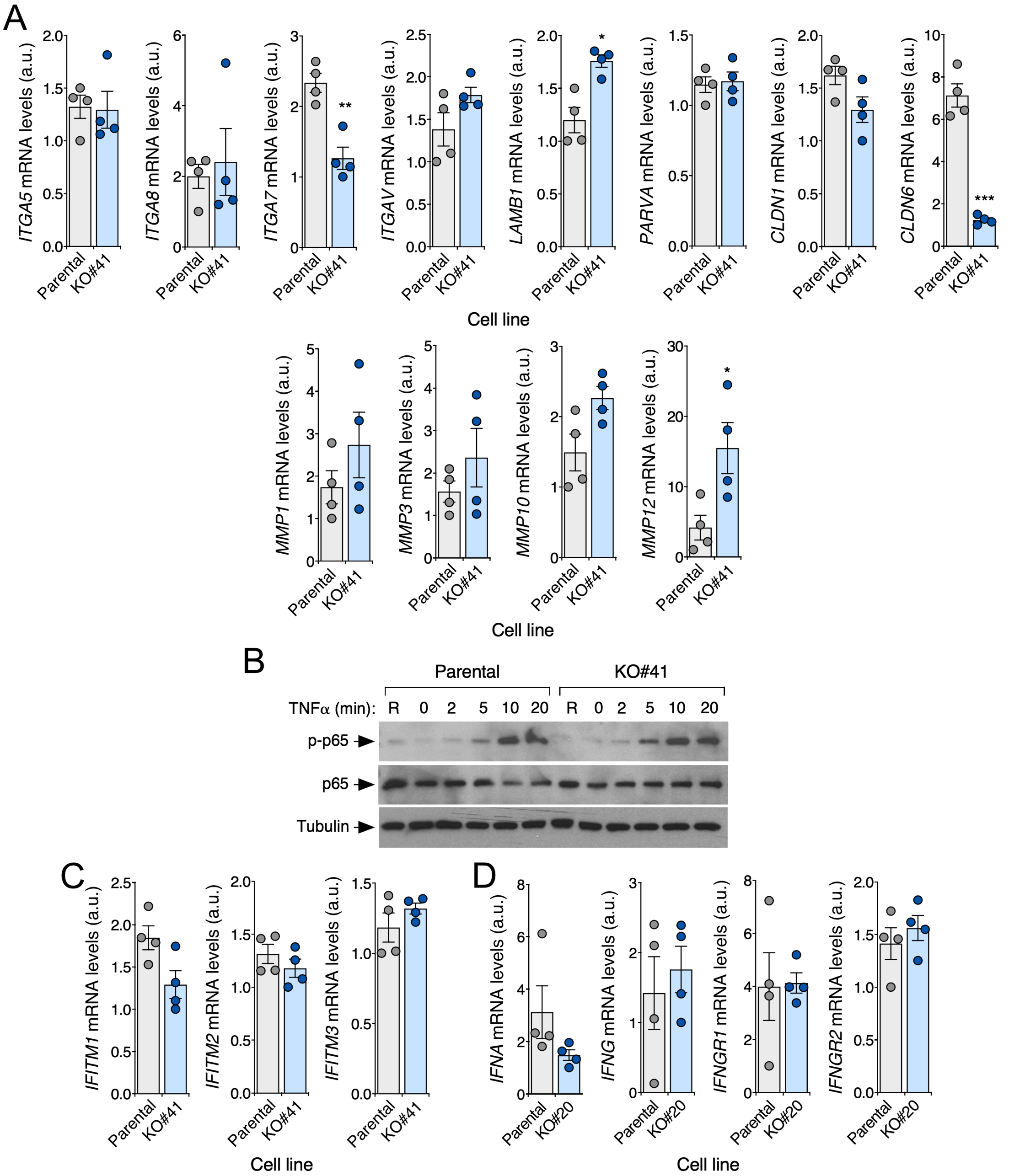

SUPPLEMENTARY FIGURE 4. Impact on the depletion of endogenous wild-type R-RAS2 in the expression of indicated genes in COV368 cells

(A) Expression levels of indicated transcripts in parental (P) and wild-type *RRAS2* knockout COV362 cells (clone KO#41) were determined using qRT-PCR analyses. Points represent independent experiments. Bars represent mean ± SEM. Values are given in arbitrary units, taking the lowest mean as 1. Statistical significance was tested by the Student's *t*-test (*, $p < 0.05$; ***, $p < 0.001$).

(B) Representative immunoblot analysis of the phosphorylation (p-) of the p65 NFκB subunit upon the stimulation of serum-starved parental and wild-type *RRAS2*-knockout COV362 cells (clone KO#41) cells with TNFα for the indicated periods of time (upper panel). As control, we evaluated the level of total p65 (middle panel) and tubulin (bottom panel) in each sample. R, resting cells. $n = 2$ independent experiments.

(C) Expression levels of indicated transcripts in parental (P) and wild-type *RRAS2* knockout COV362 cells (clone KO#41) were determined using qRT-PCR analyses. Points represent independent experiments. Bars represent mean ± SEM. Values are given in arbitrary units, taking the lowest mean as 1. Statistical significance was tested by the Student's *t*-test.

(D) Expression levels of indicated transcripts in parental (P) and *RRAS2*^{Q72L} knockout A2780 cells (clone KO#20) were determined using qRT-PCR analyses. Points represent independent experiments. Bars represent mean ± SEM. Values are given in arbitrary units, taking the lowest mean as 1. Statistical significance was tested by the Student's *t*-test.

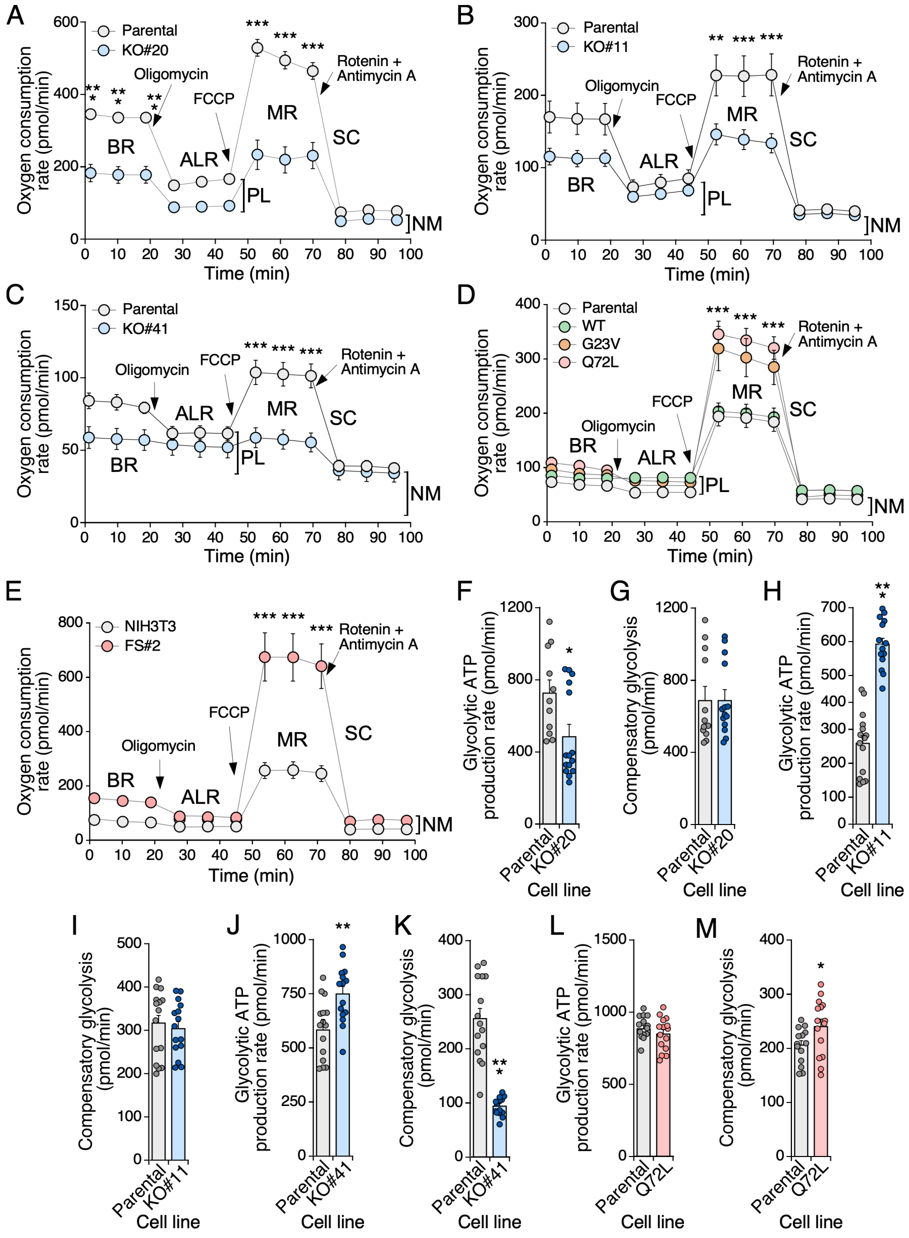

SUPPLEMENTARY FIGURE 5. R-RAS2 regulates mitochondrial activity in cancer cells

(A to E) Mitochondrial respiration profile of the *RRAS2* knockout cell lines A2780 (A), CAL-51 (B) or COV362 (C), indicated R-RAS2-expressing NIH3T3 cells (D), and the R-Ras2^{Q72}-expressing mouse fibrosarcoma cell line FS#2 (E). In E, we used NIH3T3 cells as controls. ALR, ATP-linked respiration; BR, basal respiration; MR, maximal respiration; NM, non-mitochondrial O_2 consumption; PL, proton leak; SC, spare capacity. Data represent mean ± SEM. *n* = 3 (for A and C), 4 (for B and D) and 7 (E). Statistical significance was tested by two-way ANOVA and Tukey's multiple comparisons test (**, $p < 0.01$; ***, $p < 0.001$). Due to this, the asterisks do not match those presented in Fig. 7 (which were calculated using unpaired t-test or Mann-Whitney test depending on the normality of each dataset).

(F to M) Quantitation of ATP produced by glycolysis in either the absence (F, H, J, and L) or presence (G, I, K, M) of a mitochondrial chain inhibitor in indicated A2780 (F and G), CAL-51 (H and I), COV362 (J and K), and NIH3T3 cells (L and M). Points represent individual experimental points. Bars represent mean ± SEM. Q72L, NIH3T3 cells ectopically expressing R-RAS2^{Q72L}. Statistical significance was tested by unpaired *t*-test or Mann-Whitney test, depending on the normality of the dataset (*, $p < 0.05$; **, $p < 0.01$; ***, $p < 0.001$).

SUPPLEMENTARY TABLE 4. Oligonucleotides to generate RRAS2 mutants by site-directed mutagenesis

Name	Sequence (5'to3', mutation made underlined)	Plasmid (pBlueScript SK(+))	Plasmid (pEF1/V5-HisA)
D8N RRAS2 F	GCCGGCTGGCGGAACGGCTCCGGCCAGGA	pJLR05	pJLR24
D8N RRAS2 R	TCCTGGCCGGAGCCGTTCCGCCAGCCGGC		
K14T RRAS2 F	GCTCCGGCCAGGAGACGTACCGGCTCGTGGTGGTC	pLCM1	pLCM12
K14T RRAS2 R	GACCACCACGAGCCGGTACGTCTCCTGGCCGGAGC		
G23A RRAS2 F	TGGTGGTCGGCGGGGCCGGCGTGGGCAAGT	pJLR06	pJLR25
G23A RRAS2 R	ACTTGCCCACGCCGGCCCCGCCGACCACCA		
G23C RRAS2 F	TGGTGGTCGGCGGGTGCGGCGTGGGCAAGT	pJLR07	pJLR26
G23C RRAS2 R	ACTTGCCCACGCCGCACCCGCCGACCACCA		
G23S RRAS2 F	TGGTGGTCGGCGGGAGCGGCGTGGGCAAGT	pJLR08	pJLR27
G23S RRAS2 R	ACTTGCCCACGCCGCTCCCGCCGACCACCA		
G24C RRAS2 F	TGGTCGGCGGGGGCTGCGTGGGCAAGTCG	pJLR09	pJLR28
G24C RRAS2 R	CGACTTGCCCACGCAGCCCCCGCCGACCA		
G24D RRAS2 F	TGGTCGGCGGGGGCGACGTGGGCAAGTCG	pJLR10	pJLR29
G24D RRAS2 R	CGACTTGCCCACGTCGCCCCCGCCGACCA		
G24V RRAS2 F	TGGTCGGCGGGGGCGTCGTGGGCAAGTCG	pJLR11	pJLR30
G24V RRAS2 R	CGACTTGCCCACGACGCCCCCGCCGACCA		
D42Y RRAS2 F	GTCCTATTTTGTAACGTATTATGATCCAACCATTG	pJLR12	pJLR31
D42Y RRAS2 R	CAATGGTTGGATCATAATACGTTACAAAATAGGAC		
D44E RRAS2 F	TATTTTGTAACGGATTATGAACCAACCATTGAAGAT	pLCM2	pLCM13
D44E RRAS2 R	ATCTTCAATGGTTGGTTCATAATCCGTTACAAAATA		
P45R RRAS2 F	TTGTAACGGATTATGATCGAACCATTGAAGATTC	pLCM3	pLCM14
P45R RRAS2 R	GAATCTTCAATGGTTCGATCATAATCCGTTACAA		
E48D RRAS2 F	TATGATCCAACCATTGACGATTCTTACACAAAG	pLCM4	pLCM15
E48D RRAS2 R	CTTTGTGTAAGAATCGTCAATGGTTGGATCATA		
D49Y RRAS2 F	GATCCAACCATTGAATATTCTTACACAAAGCAG	pJLR13	pJLR32
D49Y RRAS2 R	CTGCTTTGTGTAAGAATATTCAATGGTTGGATC		
K53M RRAS2 F	GAAGATTCTTACACAATGCAGTGTGTGATAGAT	pJLR14	pJLR33
K53M RRAS2 R	ATCTATCACACACTGCATTGTGTAAGAATCTTC		
R63Q RRAS2 F	GATGACAGAGCAGCCCAGCTAGATATTTTGGAT	pJLR15	pJLR34
R63Q RRAS2 R	ATCCAAAATATCTAGCTGGGCTGCTCTGTCATC		
A70T RRAS2 F	GATATTTTGGATACAACAGGACAAGAAGAGTTTG	pJLR16	pJLR35
A70T RRAS2 R	CAAACTCTTCTTGTCCTGTTGTATCCAAAATATC		

Name	Sequence (5'to3')	Plasmid (pBlueScript SK(+))	Plasmid (pEF1/V5-HisA)
Q72H RRAS2 F	GATACAGCAGGACACGAAGAGTTTGGAGCCA	pJLR17	pJLR36
Q72H RRAS2 R	TGGCTCCAAACTCTTCGTGTCCTGCTGTATC		
Q72L RRAS2 F	GATACAGCAGGACTAGAAGAGTTTGGAGCCA	pJLR18	pJLR37
Q72L RRAS2 R	TGGCTCCAAACTCTTCTAGTCCTGCTGTATC		
Y105C RRAS2 F	CAGTTTTGAAGAAATCTGTAAGTTTCAAAGACAGA	pLCM5	pLCM16
Y105C RRAS2 R	TCTGTCTTTGAAACTTACAGATTTCTTCAAAACTG		
L112I RRAS2 F	GTTTCAAAGACAGATTATCAGAGTAAAGGATCGTG	pLCM10	pLCM21
L112I RRAS2 R	CACGATCCTTTACTCTGATAATCTGTCTTTGAAAC		
R117C RRAS2 F	TCTCAGAGTAAAGGATTGTGATGAGTTCCCAATG	pLCM6	pLCM17
R117C RRAS2 R	CATTGGGAACTCATCACAATCCTTTACTCTGAGA		
R135S RRAS2 F	GATCTGGATCATCAAAGTCAGGTAACACAGGAAG	pLCM7	pLCM18
R135S RRAS2 R	CTTCCTGTGTTACCTGACTTTGATGATCCAGATC		
R147Q RRAS2 F	AGGACAACAGTTAGCACAGCAGCTTAAGGTAACA	pJLR19	pJLR38
R147Q RRAS2 R	TGTTACCTTAAGCTGCTGTGCTAACTGTTGTCCT		
A158V RRAS2 F	TACATGGAGGCATCAGTAAAGATTAGGATGAAT	pJLR20	pJLR39
A158V RRAS2 R	ATTCATCCTAATCTTTACTGATGCCTCCATGTA		
K159Q RRAS2 F	ATGGAGGCATCAGCACAGATTAGGATGAATGTA	pJLR21	pJLR40
K159Q RRAS2 R	TACATTCATCCTAATCTGTGCTGATGCCTCCAT		
M162V RRAS2 F	ATCAGCAAAGATTAGGGTGAATGTAGATCAAGCT	pLCM11	pLCM22
M162V RRAS2 R	AGCTTGATCTACATTCACCCTAATCTTTGCTGAT		
A167T RRAS2 F	ATGAATGTAGATCAAACTTTCCATGAACTTGTC	pJLR22	pJLR41
A167T RRAS2 R	GACAAGTTCATGGAAAGTTTGATCTACATTCAT		
R173W RRAS2 F	TCCATGAACTTGTCTGGGTTATCAGGAAATTTCA	pLCM8	pLCM19
R173W RRAS2 R	TGAAATTTCCTGATAACCCAGACAAGTTCATGGA		
K177T RRAS2 F	GTCCGGGTTATCAGGACATTTCAAGAGCAGGAA	pJLR23	pJLR42
K177T RRAS2 R	TTCCTGCTCTTGAAATGTCCTGATAACCCGGAC		
F204L RRAS2 F	TCCATGAACTTGTCTGGGTTATCAGGAAATTTCA	pLCM9	pLCM20
F204L RRAS2 R	TGAAATTTCCTGATAACCCAGACAAGTTCATGGA		

SUPPLEMENTARY TABLE 5. Oligonucleotides used in gene-editing

Name	Sequence (5'to 3')	Name of final plasmid (vector used)	Use
TC21 sgRNA 1 F	CACCGCGGTACCCGGGACCCCAGCC	pLCM 71 [pSpCas9(BB)-2A-GFP (PX458) (Cat. No. 48138, Addgene)]	CRISPR–Cas9-mediated *RRAS2* KO by NHEJ in A2780, COV362 and COV504 cells
TC21 sgRNA 1 R	AAACGGCTGGGGTCCCGGGTACCGC		
TC21 sgRNA 2 F	CACCGGGAGAAGTACCGGCTCGTGG	pLCM 72 [pSpCas9(BB)-2A-GFP (PX458) (Cat. No. 48138, Addgene)]	
TC21 sgRNA 2 R	AAACCCACGAGCCGGTACTTCTCCC		
TC21 sgRNA 3 F	CACCGCGCCTCGGGCGGTACCCAGC	pLCM 73 [pSpCas9(BB)-2A-GFP (PX458) (Cat. No. 48138, Addgene)]	
TC21 sgRNA 3 R	AAACGCTGGGTACCGCCCGAGGCGC		
hTC21 sgRNA 4 F	CACCGGCTCCGGCCAGGAGAAGTAC	pLCM 74 [pX330-U6-Chimeric_BB-CBh-hSpCas9 vector (Cat. No. 42230, Addgene)]	CRISPR–Cas9-mediated GFP-tagging of *RRAS2* in A2780, COV362 and CAL-51 cells
hTC21 sgRNA 4 R	AAACGTACTTCTCCTGGCCGGAGCC		
hTC21 sgRNA 5 F	CACCGGCCAGCCGGCCGCGGCCATG	pLCM 75 [pX330-U6-Chimeric_BB-CBh-hSpCas9 vector (Cat. No. 42230, Addgene)]	
hTC21 sgRNA 5 R	AAACCATGGCCGCGGCCGGCTGGCC		
hTC21 sgRNA 6 F	CACCGCTGTAGCGTCCCCATGGCCG	pLCM 76 [pX330-U6-Chimeric_BB-CBh-hSpCas9 vector (Cat. No. 42230, Addgene)]	
hTC21 sgRNA 6 R	AAACCGGCCATGGGGACGCTACAGC		
hTC21 homol left F	CTGACAAAGCTTCCCCTCGCACTCACAC TCCTCATATC	pLCM 77 [pBlueScript SK ii (+) (Cat. No. 212205, Addgene) + EGFP sequence]	Amplification of *RRAS2* left homology arm from A2780 for CRISPR–Cas9-mediated GFP-tagging of *RRAS2*
hTC21 homol left R	CCAATGGGATCCGGGGACGCTACAGAGC CCAGCC		
hTC21 homol right F	CTGACACTCGAGCTATGGCCGCGGCCGG CTGGCGGGA		Amplification of *RRAS2* right homology arm from A2780 GFP-tagging of *RRAS2*
hTC21 homol right R	CCAATGGCTAGCGCAACCAGAGAGGGCT GGGGG		
GFP polyA signal F1	CCAATGGGATCCATCGCCACCATGGTGA GCA	pLCM79	Amplification of GFP-PolyA to subclone into pLCM77. pLCM79 generates a KO while knocking in a GFP-PolyA sequence
Poly A signal R1	CTGACACTCGAGTAAGATACATTGATGA GTTTGGAC		

**Name**	**Sequence** (5'to 3')	**Name of final plasmid (vector used)**	**Use**
mTC21 sgRNA 1 F	CACCGTCGGGGCGTCCCGATGGCCG	pMFP23	CRISPR–Cas9-mediated GFP-tagging of *RrasS2* in FS#2 cells
mTC21 sgRNA 1 R	AAACCGGCCATGGGACGCCCCGAC		
mTC21 sgRNA 2 F	CACCGGCTCCGGCCAGGAGAAGTAC	pMFP24	
mTC21 sgRNA 2 R	AAACGTACTTCTCCTGGCCGGAGCC		
mTC21 homol left F	CTGACAAAGCTTCTGGGGTAAGTGCACCTTGGACCTC	pMFP29	Amplification of mouse *RRAS2* left homology arm from FS#2 cell
mTC21 homol left R	CCAATCGGATCCCGGGACGCCCCGAGCCCAG		
mTC21 homol right F	CTGACACTCGAGCTATGGCCGCGGCCGGCT		Amplification of mouse *RRAS2* right homology arm from FS#2 cells
mTC21 homol right R	CCAATCGCTAGCCGCCTTTCAGGGTGACAAAAGCAAGG		
mPAM2 mut F	GGAGAAGTACCGACTCGTGGTGGTC		Mutation of the PAM sequence for mTC21 sgRNA 2
mPAM2 mut R	GACCACCACGAGTCGGTACTTCTCC		
